# RAB5A Promotes Active Fluid Wetting by Reprogramming Breast Cancer Spheroid Mechanics

**DOI:** 10.1002/advs.202503569

**Published:** 2025-07-25

**Authors:** Grégoire Lemahieu, Paulina Moreno‐Layseca, Tobias Hub, Carlo Bevilacqua, Manuel Gómez‐González, Federica Pennarola, Federico Colombo, Andrew E. Massey, Leonardo Barzaghi, Andrea Palamidessi, Leon‐Luca Homagk, Samuel F. H. Barnett, Alexander X. Cartagena‐Rivera, Christine Selhuber‐Unkel, Robert Prevedel, Xavier Trepat, Joachim P. Spatz, Johanna Ivaska, Giorgio Scita, Elisabetta Ada Cavalcanti‐Adam

**Affiliations:** ^1^ Department of Cellular Biophysics Max Planck Institute for Medical Research Jahnstraße 29 69120 Heidelberg Germany; ^2^ Turku Bioscience Center University of Turku, Åbo Akademi University Tykistökatu 6 Turku 20520 Finland; ^3^ Cell Biology, Biophysics European Molecular Biology Laboratory (EMBL) Meyerhofstraße 1 69117 Heidelberg Germany; ^4^ Institute for Bioengineering of Catalonia (IBEC) The Barcelona Institute for Science, Technology (BIST) C/ Baldiri Reixac 10–12 Barcelona 08028 Spain; ^5^ Departament de Genètica Microbiologia i Estad ística Facultat de Biologia Universitat de Barcelona Barcelona Spain; ^6^ Institute for Molecular Systems Engineering, Advanced Materials (IMSEAM) Heidelberg University 69120 Heidelberg Germany; ^7^ Section on Mechanobiology National Institute of Biomedical Imaging, Bioengineering National Institutes of Health South Drive 50 Bethesda MD 20892 USA; ^8^ Mechanisms of Tumor Cell Migration IFOM ETS – The AIRC Institute of Molecular Oncology Via Adamello 16 Milan 20139 Italy; ^9^ Department of Oncology, Haemato‐Oncology University of Milan Via Festa del Perdono 7 Milan 20122 Italy; ^10^ German Center for Lung Research (DZL) Im Neuenheimer Feld 156 69120 Heidelberg Germany; ^11^ Centro de Investigación Biomédica en Red en Bioingenería Biomateriales y Nanomedicina (CIBER‐BBN) Bellaterra Barcelona 08193 Spain; ^12^ Facultat de Medicina Universitat de Barcelona Carrer de Casanova 143 Eixample Barcelona 08036 Spain; ^13^ Institució Catalana de Recerca i Estudis Avançats (ICREA) Passeig Lluís Companys 23 Barcelona 08010 Spain; ^14^ Department of Life Technologies University of Turku Turku 20014 Finland; ^15^ InFLAMES Research Flagship Center University of Turku Turku 20014 Finland; ^16^ Foundation for the Finnish Cancer Institute Tukholmankatu 8 Helsinki 00014 Finland; ^17^ Cellular Biomechanics Faculty of Engineering Sciences Bayreuth University Universitätsstraße 30 95447 Bayreuth Germany

**Keywords:** adhesion, biophysics, mechanobiology, RAB5A‐mediated breast carcinoma fluidification, softening, spheroid wetting, supracellular motility

## Abstract

Unjamming transitions from a solid‐like to a fluid‐like state are a gateway to breast epithelial cancer invasion. However, the mechanical interplay between phase transitions and dimension transitions, in particular wetting, remains elusive, despite being critical for understanding the onset of metastatic dissemination. This study shows that unjamming, mediated by the RAB5A GTPase, alters carcinoma spheroid fluidity, rigidity, and rewires adhesion mechanics to drive supracellular active wetting as a new mode of tumor expansion. Spheroid fluidification enhances the selective expression of integrin subunits and increases focal adhesion dynamics, inducing a fluid‐like spreading behavior on specific matrix ligands. Notably, nanoscale regulation of integrin clustering can select for distinct phase transitions at the collective scale upon wetting. In this framework, fluidized spheroids polarize into cohesive “supracells”, and maintain a stiff peripheral actin bundle as measured by nanomechanical mapping. Furthermore, a combination of Brillouin microscopy and 2.5D traction force analysis reveals a mechanical switch within the spheroid core, characterized by significant cell softening and a reduction in compressive forces exerted on the substrate, thereby mimicking the wetting of a liquid droplet. These findings establish unjamming‐driven active wetting as a key mechanism to comprehend the molecular and biophysical underpinnings of solid tumor invasion.

## Graphical abstract

RAB5A‐mediated unjamming enhances active wetting through distinct biomechanical processes: a) upregulation of specific integrin subunits, enhanced focal adhesions kinetics and activity; b) supracellular organization relying on nanoscale integrin clustering, and the formation of a stiff and collective actin cable; and c) softening of the spheroid core driving the escape from a jammed state, and decreased compressive stress exerted on the substrate.

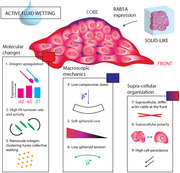

## 1 Introduction

In both physiological and pathological conditions, epithelial cell collectives undergo transition states dictated by changes in their mechanical properties.^[^ ^1^, ^2^ ^]^ In vivo and in vitro studies have shown that mature epithelial sheets typically evolve into a static, rigid jammed state. However, these sheets can become unjammed, or fluid‐like, in response to changes in cell density, motility, adhesion.^[^ ^3^ ^]^

In breast epithelial cancer, jamming‐to‐unjamming transition is thought to be a gateway to tumor dissemination.^[^ ^4^, ^5^, ^6^ ^]^ In particular, the invasive behavior of ductal carcinoma in situ (DCIS) is linked to the expression of the endosomal protein RAB5A, which is associated with poor prognosis and reduced survival of breast cancer patients.^[^ ^7^ ^]^ At the molecular level, RAB5A increases the internalization of epidermal growth factor receptor, leading to increased activation of ERK1/2 kinase—an axis which drives the formation of actin‐based structures, including lamellipodia and dorsal ruffles.^[^ ^7^, ^8^, ^9^ ^]^ Additionally, RAB5A influences the trafficking and signaling of cell surface receptors critical for collective migration, such as integrins and cadherins. Yet, the role of adhesion mechanics in this transition process remains to be understood, particularly with respect to the coordinated regulation of cell‐cell and cell‐matrix dynamics that drive invasive behavior.

Carcinoma fluidification has been shown to promote motility and invasion at a collective scale. In dense, 2D kinetically arrested epithelia, RAB5A expression induces a reawakening of collective motility characterized by long‐range, ballistic cell trajectories and local cellular rearrangements reminiscent of flocking‐fluid motion.^[^ ^10^, ^11^ ^]^ In a 3D state, RAB5A‐expressing spheroids exhibit persistent, coherent rotational motion, and remodel the extracellular matrix (ECM), leading to enhanced invasiveness.^[^ ^8^ ^]^ Thus, RAB5A expression in both 2D and 3D cell collectives triggers a phase transition from a glassy, solid‐like state to a hyper‐motile, flocking‐fluid phase that promotes the conversion from confined into invasive breast carcinoma. However, as malignant dimension shifts and phase transitions can be entangled in tissues, the mechanobiology behind RAB5A expression driving changes in dimensions (3D to 2D) for multicellular systems remains unclear.

As a shift from a 3D structure to a 2D expanding monolayer, the wetting of multicellular aggregates can account for alterations in tissue mechanical properties.^[^ ^12^ ^]^ First described as a competition between cell‐substrate and cell‐cell adhesion energies like for fluid drops,^[^ ^12^, ^13^, ^14^, ^15^ ^]^ spheroid wetting has recently been modeled as an active mechanism relying on a balance between traction forces, contractile intercellular stresses, and surface tension.^[^ ^16^, ^17^ ^]^ In this framework, the physical state of the cell aggregate should impact the wetting behavior, as it relates to the viscosity through contractility, cell‐cell contacts dynamics, and cell density. In particular, the core of the spheroid reaches a jammed, solid‐like state upon cell aggregation and increase in cell density, yet it behaves as a fluid system at long times. As a consequence of the overall surface tension of the aggregate, the spheroid core exerts out‐of‐plane compressive forces (or negative tractions) on the substrate, when the edge exerts positive, upward‐directed tractions.^[^ ^17^ ^]^ We posited that adhesion strength, spheroid rigidity, and the mechanical (de)coupling between the spheroid edge and core might be altered upon cancer fluidification.

We address this hypothesis by examining the mechanical behavior of MCF10.DCIS.com cell spheroids spreading on matrix‐coated substrates. These cells express the oncogenic variant T24‐H‐RAS and provide a model for the progression of DCIS toward invasive ductal carcinoma (IDC),^[^ ^18^ ^]^ and were engineered to express RAB5A protein in a doxycycline‐inducible manner to levels similar to those found in aggressive human breast tumors.^[^ ^8^, ^10^, ^19^ ^]^ This model system simulates some of the features observed in the progression of in situ breast carcinoma to invasive carcinoma. Using RAB5A‐mediated unjamming coupled to a comprehensive multiscale approach, we scrutinize the regulation of adhesion and the mechanical forces that facilitate the wetting by promoting a fluid‐like state in the spheroids. Our findings indicate that supracellular coordinated mechanical regulation between the spheroid core and periphery is essential for fluid‐like wetting.

## 2 Results

### 2.1 RAB5A Expression Induces Fast and Fluid‐Like Spheroid Wetting

**Figure 1:**
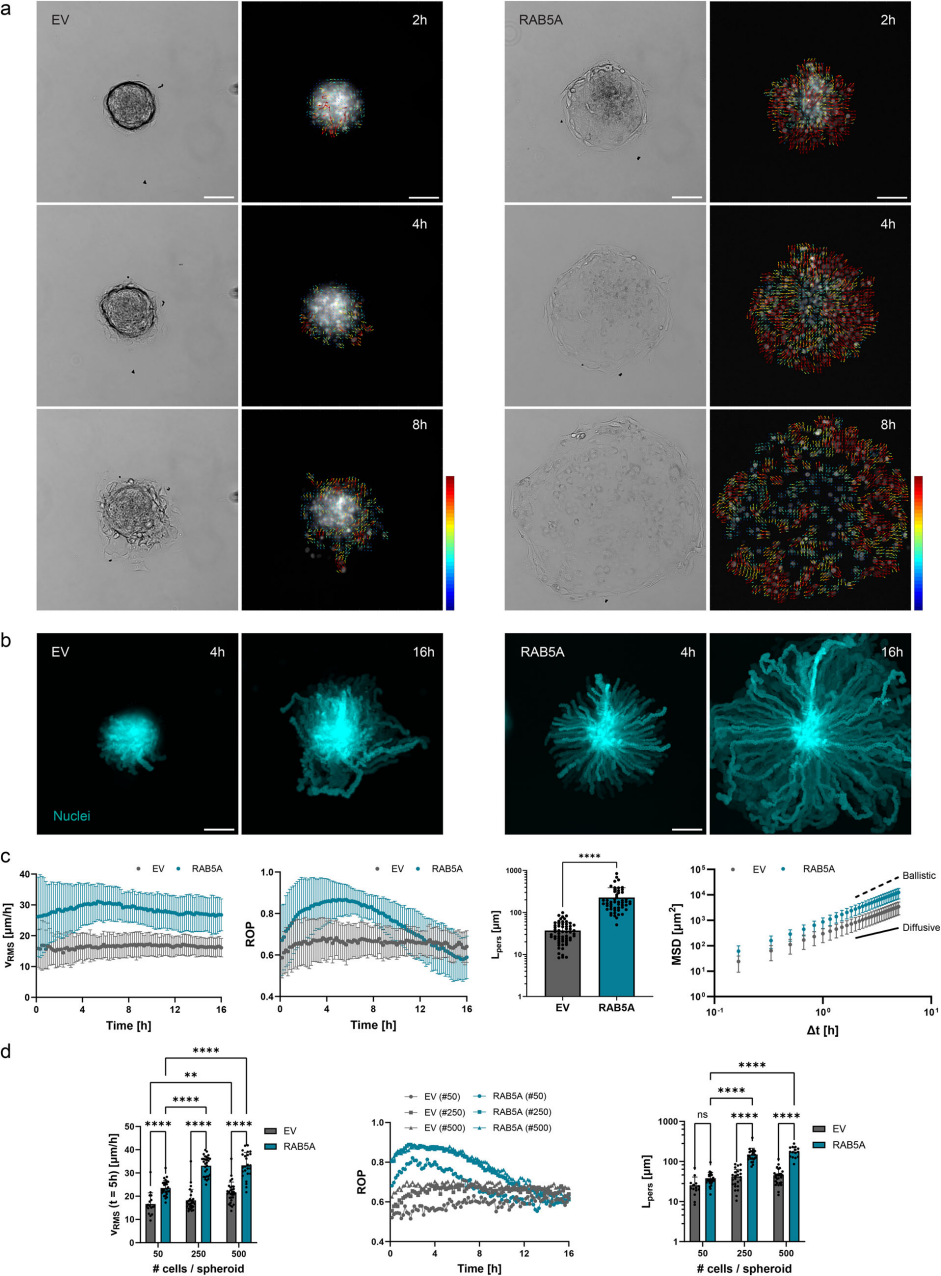
High level of RAB5A expression promotes spheroid total wetting on matrix‐coated surfaces. a) Time‐lapse brightfield images and quiver plots from particle image velocimetry (PIV) analysis on nuclei movement during empty vector (EV) control (left panel) and RAB5A‐expressing (right panel) spheroid wetting on fibronectin‐coated glass substrates. Scale bar, 100 µm. Quiver plot color bar maximum speed, 30 µm h^−1^ (dark red). b) Maximum projections of nuclei time‐lapse images during EV (left panel) and RAB5A‐expressing (right panel) spheroid wetting. Scale bar, 100 µm. c) Collective metrics computed from PIV analysis on nuclei movement of EV and RAB5A‐expressing uniformly sized spheroids (250 cells per spheroid at seeding) spreading on fibronectin‐coated glass substrates: *V*_RMS_, root mean square velocity; ROP, radial order parameter; *L*_pers_, persistence length; MSD, mean square displacement. Data are the mean with SD, *n* = 6 independent experiments (*V*_RMS_, ROP, *L*_pers_) or *n* = 3 independent experiments (MSD). ^****^p < 0.0001, Mann‐Whitney test. d) Collective metrics computed from PIV analysis on nuclei movement of EV and RAB5A‐expressing spheroids of different sizes spreading on fibronectin‐coated glass substrates: *V*_RMS_ [*t =* 5h], root mean square velocity at *t* = 5h after spheroid seeding on substrate. Data are the mean with SD (*V*_RMS_ [*t =* 5h], *L*_pers_) or the mean (ROP), *n* = 3 independent experiments. ^**^p < 0.01, ^****^p < 0.0001, *ns* not significant, two‐way analysis of variance (ANOVA) multiple comparisons.

Spheroids with a solid‐like character are less invasive and consistently display slower cell spreading rates during wetting.^[^ ^16^ ^]^ Here, the elevated expression of RAB5A was sufficient to remarkably accelerate collective spheroid wetting on fibronectin‐coated substrates (**Figures** **1**a and S1a,b and Movie S1.1, Supporting Information). This was corroborated by increased values of cell velocity as measured by particle image velocimetry (PIV) analysis of single‐cell fluorescent nuclei at the spheroid surface boundary (Figure 1a). In contrast, empty vector (EV) control spheroids underwent partial wetting and showed frequent events of cell detachment (Figure 1a). Qualitatively, in both control and RAB5A‐expressing spheroids, cells exhibited a velocity gradient from the spheroid core toward the leading front, indicating the onset of a polarized velocity flow at the multicellular scale (Figure 1a). Nuclei trajectories showed (outward) radial movements for RAB5A‐expressing cells over several hours, whereas control cells displayed less directed motion between 4 and 16 h after the wetting onset (Figure 1b and Movie S1.2, Supporting Information). Quantitative measurements of nuclei collective dynamics by PIV analysis documented a significant and robust increase in root mean square velocity (*V*_RMS_), radial order parameter (ROP), and persistence length (*L*_pers_) in RAB5A‐expressing spheroids (Figure 1c). In particular, the *V*_RMS_ curve showed a fast velocity increase upon RAB5A induction in the early spreading phase up to an average of 30 µm h^−1^, followed by a slow but steady decrease in cell speed as the spheroid transforms into a 2D monolayer. These results suggest that one or more critical factors are driving the transition at the onset of the spreading phase. Specifically, the increased rate of outward‐directed movement observed in RAB5A‐expressing cells during the first 12 h indicates a tendency for cells to move away from the spheroid core, with this outward motion peaking between 2 and 6 h after seeding time. Following this initial phase, we observed a behavioral shift characterized by either random or inward‐directed movement (ROP ≤ 0.5). This change indicates that at this point, all cells from the original 3D structure established contact with the substrate, leading to a spatial transition in their movement patterns. To investigate nuclei motion directionality, we calculated the persistence length, which measures the average distance a cell travels in a straight line before its direction of movement becomes randomized. This was assessed over a period from 1 to 12 h after the onset of spreading. The persistence length increased significantly from a mean value of 37 µm for EV spheroids to 227 µm for RAB5A‐expressing spheroids, indicating that upon RAB5A expression, cells traveled a distance greater than 11 cell diameters before switching to a more erratic motion. This substantial increase in persistence length highlights the enhanced directional movement of RAB5A‐expressing cells compared to the control, suggesting a more efficient spreading. In this perspective, the evolution of the mean square displacement (MSD) of the cell nuclei follows a power law relationship with time *MSD*  ≈  Δ*t* ^ν^, with ν  =  2 for a ballistic regime, ν  =  1 for a diffusive type of motion. The curves for both EV, RAB5A‐expressing spheroids exhibit a super‐diffusive regime—with an exponent ν comprised between 1 and 2—although the slope for the latter case is slightly higher, suggesting a more directional movement upon RAB5A induction (Figure 1c).

Our framework for spheroid spreading builds on concepts from passive systems like liquid, viscoelastic droplets, but incorporates active forces typical of cell assemblies. To support this, we acquired both bottom‐view and side‐view data to compare DCIS spheroid spreading with classical and active wetting behaviors.

The contact area growth of spheroids over time has been modeled based on the balance between interfacial energies (substrate‐medium, spheroid‐medium, spheroid‐substrate). Specifically, it has been reported that the power law fit represented as *S*(*t*) = α.*t* ^β^—where *S* is the spheroid spreading area, *t* is time, *α, β* are fitted parameters—is characterized in the early spreading stage by an exponent β  =  2/3, where cell aggregates are modeled as soft viscoelastic polymeric beads, while later wetting stages tend to follow a power law of exponent β  =  1, i.e. a diffusive regime like for simple liquids.^[^ ^15^, ^20^ ^]^ Thus, the growth of the contact area provides a useful proxy for inferring to some extent viscous or fluid‐like behavior at the tissue level. In our DCIS model, control spheroids displayed sublinear area growth (2–10 h), indicative of a viscous‐like regime (Figure S1c,d, Supporting Information), while RAB5A‐expressing spheroids followed an almost linear trend (*β* ≈1), consistent with fluid‐like spreading. Control spheroids also transitioned to linear spreading, though on a delayed timescale. Interestingly, after 10 h, *β *decreased in RAB5A‐expressing spheroids, likely reflecting constraints from monolayer tension and cell‐cell viscosity once full substrate contact is established. Similar biphasic spreading behavior has been observed at the single‐cell level, with early diffusive power‐law dynamics (the average cell contact radius *R* increases with time *t* as *t* ^1/2^) followed by slower, sub‐diffusive spreading (*R*∝*t* ^1/4^).^[^ ^21^ ^]^ We posit that a similar mechanism might be at play in the late spreading phase of RAB5A‐expressing cell spheroids.

To support the above‐mentioned bottom‐view quantification, we acquired side‐view time‐lapse images of spheroid nuclei and membrane. Spheroids displayed a bulk/core region which extends over time into a wedge, and further into a thin cell layer at the migrating edge, although with strikingly different dynamics for control or RAB5A‐expressing samples (Figure S1e and Movies S1.3 and S1.4, Supporting Information). Qualitatively, the shape of control spheroids at 3 and 5h resembled the one of polymer melts, comprised of a spherical cap, a foot, and a precursor film region, thus highlighting the effect of viscosity during DCIS spheroid spreading.^[^ ^13^ ^]^ The dynamical contact angle θ_*D*_, measured at the interface between the core and the solid substrate—to reflect the balance of interfacial tensions for the bulk aggregate^[^ ^13^, ^22^ ^]^—qualitatively decreased faster upon RAB5A expression, as compared to the control condition. Upon RAB5A induction, the apparent contact angle which vanished as the droplet flattened is a signature of active wetting.^[^ ^23^ ^]^ The side‐view images also qualitatively captured the sharper decrease of the aspect ratio (height/width) of RAB5A‐expressing spheroids over time, in respect to EV aggregates. This evolution of the aspect ratio has been rationalized using active stress corresponding to a force dipole density. For extensile active droplets, higher activity increases the contact area with the substrate and lowers the apparent contact angle, thereby decreasing the aspect ratio. In this framework, the height profile of control spheroids matches representations of spread contractile drops in the partial wetting regime, being higher and fatter than for spread extensile drops, which are flat on top with precursor regions of quadratic variations at the edges.^[^ ^24^ ^]^ The latter resembles the height profile of RAB5A‐expressing spheroids over time, and hints toward a different interplay between active stresses and surface tension than in the control case (Figure S1e, Supporting Information). These first semi‐quantitative observations suggest that RAB5A‐expressing spheroids are less viscous but more fluid‐like instead, and exhibit higher activity than the control case, thereby highlighting the role of RAB5A in enhancing the dynamics of cell spreading and invasion.

Next, we considered spheroid size as another determinant of wetting.^[^ ^15^ ^]^ To further explore its influence during the spreading, we generated monodisperse batches of spheroids with varying diameters by adjusting the cell seeding density in microwell plates (see Experimental section). We observed significant differences in *V*_RMS_ and ROP for both EV and RAB5A‐expressing aggregates as a function of the spheroid size, with in the latter case a behavioral plateau at seeding densities of 250 and 500 cells per spheroid (Figure 1d). In small spheroids, the pronounced decrease in persistence length upon RAB5A expression is likely due to limited maximal extent of the spheroid. Consistent with previous studies, we also scored increased wetting velocity as a function of the spheroid size in both control and RAB5A‐expressing cells (Figure S1f, Supporting Information).^[^ ^15^ ^]^

Taken together, these findings suggest that RAB5A drives a complete and fluid‐like type of spheroid wetting.

### 2.2 Elevated Level of RAB5A Increases Expression of Specific Integrins

Next, we sought to determine whether this enhanced spreading was a result of altered cell‐substrate interactions following RAB5A induction. In this perspective, αβ‐integrin heterodimers serve as crucial connections between the ECM and the cell's actin cytoskeleton,^[^ ^25^ ^]^ through a group of mechanosensitive intracellular focal adhesion (FA) proteins, which link the integrins to the cytoskeleton enabling them to act as “molecular clutches”.^[^ ^26^, ^27^ ^]^ The dysregulation of integrin‐mediated adhesion is often a sign of malignancy.^[^ ^28^, ^29^ ^]^

**Figure 2:**
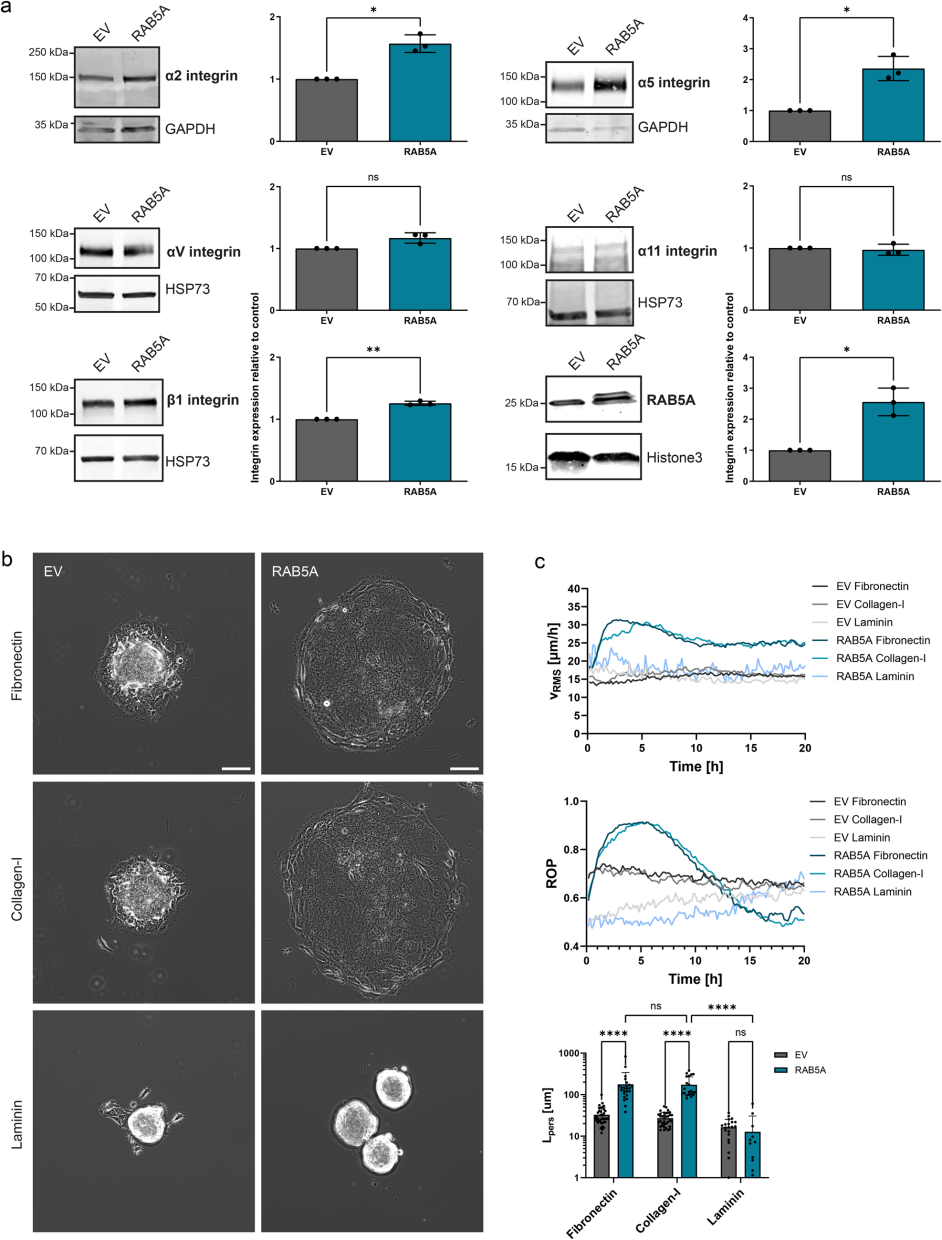
RAB5A‐mediated wetting positively regulates specific integrin pools. a) Western blot images andquantifications of integrin subunit protein levels in control and RAB5A‐expressing hyperconfluent cell monolayers 6h after the second addition of doxycycline. Data are the mean with SD, *n* = 3 independent experiments. ^*^p < 0.05, ^**^p < 0.01, *ns* not significant, paired *t*‐test. b) Time‐lapse brightfield images of EV (left) and RAB5A‐expressing (right) spheroids wetting on matrix‐coated glass substrates: fibronectin (top), collagen‐I (middle), and laminin‐rich (bottom). Timepoint is 6 h after spheroid seeding. Scale bar, 100 µm. c) Collective metrics computed from PIV analysis on nuclei movement of EV and RAB5A‐expressing spheroids wetting on matrix‐coated glass substrates. Data are the mean (*V*_RMS_, ROP) or the mean with SD (*L*_pers_), *n* = 3 independent experiments. ^****^p < 0.0001, *ns* not significant, two‐way ANOVA multiple comparisons.

To determine which integrins are involved in this process, we induced cell streaming motility in otherwise‐jammed DCIS monolayers via RAB5A induction and examined protein levels of different integrin subunits by western blotting (**Figure** **2**a). We found that integrin subunits α2, α5, and β1 were significantly upregulated upon RAB5A expression. These integrin subunits can be upregulated in metastatic cancer cells and are associated with higher drug survival as well as enhanced migration and tumor invasion.^[^ ^30^, ^31^, ^32^ ^]^ Integrin subunits αV and α11, instead, showed a high expression level but no significant difference when comparing control and RAB5A‐expressing cells. Of note, laminin‐binding integrin subunits (α3, α6, β4) showed no or very low expression in 2D monolayers.

The specific integrin heterodimers upregulated upon RAB5A expression are expected to trigger distinct adhesions to ECM proteins, with α5β1 integrin acting as a fibronectin and α2β1 integrin as a collagen receptor. To test this hypothesis, we performed spheroid wetting assays on fibronectin, collagen‐I, and laminin from Engelbreth‐Holm‐Swarm (EHS) murine matrices. We observed that RAB5A‐expressing aggregates undergo a fluid‐like wetting on both fibronectin‐ and collagen‐I‐coated glass substrates, but not on laminin‐rich surfaces (Figure 2b and Movies S2.1–S2.3, Supporting Information). On laminin‐rich substrate, both EV, RAB5A‐expressing spheroids showed poor ECM adhesion, remained highly cohesive and round over time, with occasional cell detachment from the core, reminiscent of a solid‐to‐gas type of transition (Figure 2b). As expected, high expression levels of α5β1 and α2β1 integrins translated to enhanced ECM engagement on fibronectin‐ and collagen‐I‐rich substrates, while low expression of α3 and α6 integrins prevented the spreading on laminin‐rich substrates.^[^ ^33^, ^34^, ^35^ ^]^ *V*_RMS_, ROP curves showed similar, reproducible trends for spheroids spreading on fibronectin and collagen‐I substrates (Figure 2c). Interestingly, ROP curves for RAB5A‐expressing cells on laminin‐rich matrices showed late spreading outside of the core, possibly owing to cell‐derived ECM deposition, or to engagement of a different pool of integrins over time. Upon RAB5A induction, persistence length values reached 178 µm on fibronectin substrates, and 173 µm on collagen‐I surfaces with no significant difference (Figure 2c). In contrast, spheroid spreading on laminin ligands was characterized by a low persistence for both control and RAB5A‐expressing cells (Figure 2c).

We also investigated whether this active wetting is driven by increased ECM production upon RAB5A induction. To this end, we quantified the protein expression level of fibronectin using Western blot analysis during RAB5A‐mediated cell streaming assay. Notably, we did not observe any significant differences in fibronectin protein expression compared to the control (Figure S2a, Supporting Information).

Collectively, these findings indicate that RAB5A selectively enhances specific pools of integrin heterodimers that allow for selective spreading behaviors. Thus, we aimed to examine how the recruitment of integrins translates into FA dynamics during the wetting.

### 2.3 Spheroid Fluidification Promotes Focal Adhesion Kinetics and Drives Supracellular Wetting

Mechanotransduction is the process by which cells sense the biophysical properties of a substrate and show adaptation to the extracellular milieu.^[^ ^36^ ^]^ For cells to migrate, FAs must be dynamic and translate traction forces exerted on the substrates into biochemical cell signals.^[^ ^37^ ^]^ Their dynamics are facilitated by the rapid endocytic and exocytic trafficking of integrins, which is often dysregulated in cancer.^[^ ^38^, ^39^ ^]^ As RAB5A is responsible for the recycling of specific cargo to the cell plasma membrane, including integrin subunits, we asked whether fluid‐like wetting displays altered FA dynamics.

**Figure 3:**
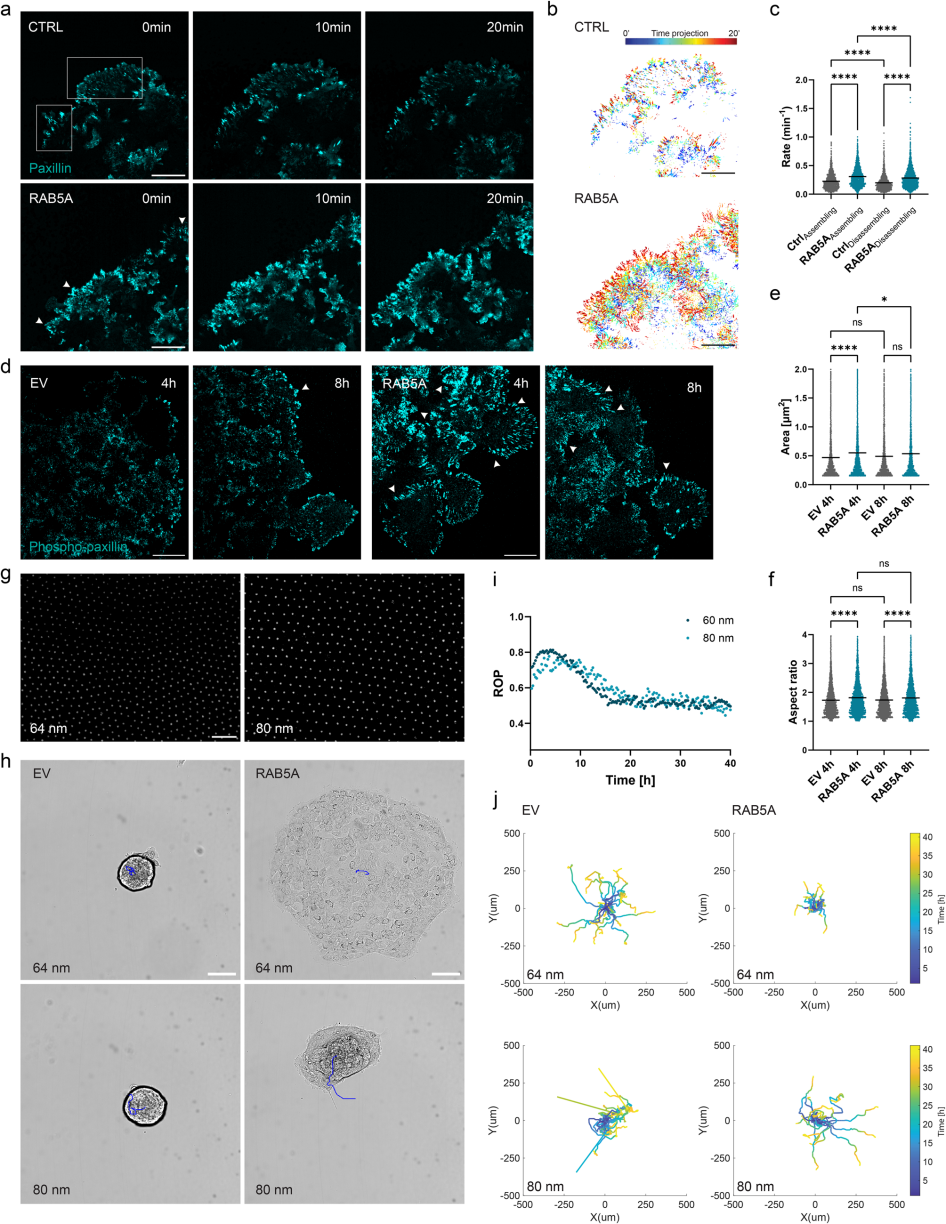
RAB5A‐mediated spheroid fluidification increases focal adhesion dynamics and fosters a supracell‐like wetting. a) Time‐lapse images of live paxillin‐stained control (top) and RAB5A‐overexpressing (bottom) spheroids wetting on fibronectin‐coated glass substrates. Rectangles show regions of slower focal adhesion kinetics, while arrows point toward dynamic adhesion clusters and lamellipodia. Scale bar, 25 µm. b) Ghost images of the focal adhesions shown in (a). Scale bar, 25 µm. c) Rate of focal adhesions assembly and disassembly in control and RAB5A‐expressing spheroids. Data are scatter plots with mean, *n* = 3 independent experiments. ^****^p < 0.0001, two‐way ANOVA with multiple comparisons. d) Phosphopaxillin immunostaining images of EV (left panel) and RAB5A‐expressing (right panel) spheroids wetting on fibronectin‐coated glass substrates. Arrows point toward high aspect ratio, large adhesion clusters. Scale bar, 25 µm. e) Cross‐sectional area of phosphopaxillin‐positive clusters. Data are scatter plots with mean, *n* = 3 experiments. ^*^p < 0.05, ^****^p < 0.0001, *ns* not significant, Kruskal‐Wallis test with multiple comparisons. f) Aspect ratio of phosphopaxillin‐positive clusters. Data are scatter plots with mean, *n* = 3 experiment. ^****^p < 0.0001, *ns* not significant, Kruskal‐Wallis test with multiple comparisons. g) Scanning electron microscopy (SEM) images of block copolymer micellar nanolithography (BCMN) surfaces. Gold nanoparticle spacings are around 64 nm (left) and 80 nm (right). Scale bar, 200 nm. h) Time‐lapse images of EV (left) and RAB5A‐expressing (right) spheroids wetting on nanopatterned surfaces with 64‐nm (top) and 80‐nm (bottom) adhesive ligands spacing. Timepoint is 20 h after spreading onset. Scale bar, 100 µm. i) Radial order parameter (ROP) of RAB5A‐expressing spheroids spreading on BCMN substrates. Data are the mean, *n* = 2 independent experiments. j) Centre‐of‐mass displacement over time of EV (left) and RAB5A‐expressing (right) spheroids wetting on BCMN substrates. Ligand spacings are around 64 nm (top) and 80 nm (bottom), *n* = 2 independent experiments.

We visualized the kinetics of FA assembly and disassembly in MCF10.DCIS‐HA‐RAB5A cells expressing green fluorescent protein (GFP)‐paxillin. FAs were recorded every 30 s for 20 min, in a timespan ranging from 2 to 10 h after spheroid seeding on fibronectin‐coated glass substrate in doxycycline‐ or vehicle‐treated cells. Compared to the control, RAB5A‐expressing cells exhibited highly dynamic lamellipodia at the leading edge (**Figure** **3**a,b and Movie S3.1, Supporting Information). Overall, the segmentation analysis of FAs over time revealed that RAB5A induction resulted in significantly faster rates of assembly and disassembly, with increases of 36% and 41%, respectively, compared to control values (Figure 3c).^[^ ^40^ ^]^ These results highlight the pivotal role of RAB5A in controlling FA turnover, presumably through fast endo‐, exocytic cycles of FA key components. Of note, we found that the rates of disassembly are slightly but significantly lower than the assembling kinetics for both cell conditions (Figure 3c). To further explore the structural evolution of FAs, we next fixed spheroids after 4 and 8h of spreading motility to assess phosphopaxillin clustering (Tyr 118), given that tyrosine phosphorylation of paxillin increases both FA assembly, turnover, and lamellipodial protrusions.^[^ ^41^, ^42^ ^]^ Interestingly, we found that the phosphopaxillin‐containing adhesions were longer at both timepoints upon RAB5A induction, while the control cells displayed mostly grain‐like complexes (Figure 3d–f). More specifically, larger peripheral cell layers exhibited outward‐directed, elongated complexes enriched with phosphorylated paxillin upon RAB5A expression, as compared to the control condition, thereby suggesting a higher degree of clustering.

In summary, these observations show that RAB5A enhances spheroid adhesion dynamics and highlight a specific form of unjamming driven by increased cell‐substrate interactions.

Adhesion strength and dynamics are key regulators of cell migration. It was previously shown that clustering is critical for force distribution at integrin binding sites,^[^ ^43^ ^]^ and in single cells, stable formation of FAs and persistent spreading depend on ligand density.^[^ ^44^ ^]^ Notably, single cell motility is enhanced on large ligand spacing (≥ 80 nm) but is less persistent compared to spacing in the range of 50–60 nm. Therefore, we investigated the impact of the expression of RAB5A on adhesion strength by manipulating integrin lateral clustering during the active wetting. We employed BCMN to create hexagonal arrays of cyclic arginine‐glycine‐aspartic acid (cRGD)‐biofunctionalized gold nanoparticles, which were surrounded by a cell‐repellent polyethylene glycol (PEG) coating in the gaps of the nanolattice. This nanopatterning technique enabled us to modulate integrin clustering by adjusting the spacing between the nanoparticles onto the substrate.^[^ ^45^ ^]^ We observed that EV control spheroids seeded on BCMN surfaces with a 64‐nm‐spaced pattern undergo a solid‐to‐gas transition, with single cells detaching from the aggregate core, whereas RAB5A expression was still able to drive a solid‐to‐liquid transition as previously seen on fibronectin‐coated surfaces (Figure 3g,h and Movie S3.2, Supporting Information). Interestingly, increasing the spacing between adhesive ligands to 80 nm hindered the wetting behavior upon RAB5A induction (Figure 3h), but allowed them to still migrate spread in a collective manner. Consistent with these observations, we observed a delayed, less (outward) radial spreading on 80‐nm‐spaced pattern upon RAB5A expression, as compared to lower spacing (Figure 3i). We further quantified the displacement of the center‐of‐mass of the cell aggregates and showed that RAB5A‐expressing spheroids are more motile on large ligand spacing (Figure 3j). These findings suggest that tissue fluidification can replicate the adhesion and, spreading behaviors—directly linked to integrin clustering effects—of single cells within multicellular aggregates. Consequently, RAB5A‐driven wetting, exhibits the characteristics of supracellular spreading.^[^ ^46^ ^]^

The previous results highlight the impact of RAB5A expression on focal adhesion dynamics. RAB5A not only increases the kinetics of assembly and disassembly of FAs, but also promotes the accumulation of active FAs necessary for long‐range transmission of cellular forces. Moreover, RAB5A‐expressing spheroids recapitulate the single cell spreading behavior observed when varying integrin lateral clustering.

### 2.4 A Core‐Edge Polarization Governs the RAB5A‐Mediated Collective Wetting

Next, we sought to determine which are the drivers of tissue spreading at the front and at the core of the cell collective. Supracellular migration depends on long‐range cytoskeletal organization and cell polarization.^[^ ^47^ ^]^ In contrast to single‐cell motility, collective migration requires the preservation of cell‐cell contacts which provide cohesion and mechanical coupling during movement.^[^ ^48^ ^]^ Cell‐cell connections coordinate multicellular movement by establishing cooperative actin dynamics, and transmitting traction forces. While the cell junction dynamics have been studied upon RAB5A expression, mostly in dense monolayers,^[^ ^8^, ^10^ ^]^ the forces exerted by the edge and core cell cohorts migrating in free space, and their mechanical coupling, are largely unexplored. Therefore, we set out to investigate the establishment of a polarization axis during the wetting.

**Figure 4:**
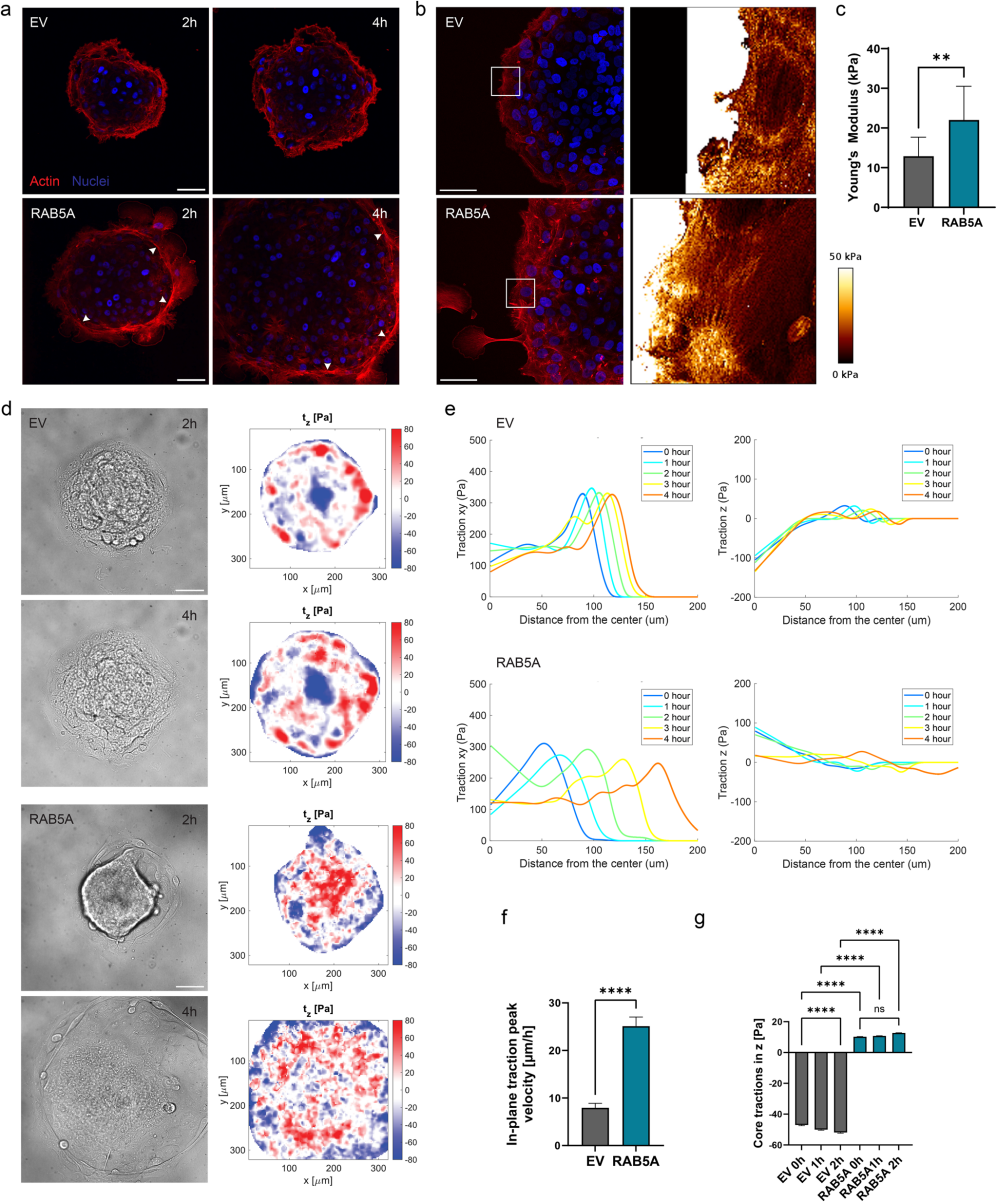
RAB5A‐mediated spheroid wetting relies on core‐edge polarization. a) Time‐lapse images of live actin (SPY650‐FastAct) and nuclei staining in EV (top), RAB5A‐expressing (bottom) spheroids wetting on fibronectin‐coated glass substrates. Images were denoised using the NIS software analysis. Arrows point toward the supracellular actin cable continuously shared by the front migratory cells. Scale bar, 50 µm. b) Atomic force microscopy nanomechanical mapping of the peripheral actin structures in EV control (top) and RAB5A‐expressing (bottom) spheroids 6h after seeding on fibronectin‐coated glass substrates. AFM maps displayed correspond to the regions drawn in the nuclei/actin fluorescent images. Scale bar, 50 µm. c) Average Young's modulus of the peripheral actin cable in EV and RAB5A‐expressing spheroids. Data are the mean with SD, *n* = 2 independent experiments. ^**^p < 0.01, Mann‐Whitney test. d) Time‐lapse brightfield images (left) and out‐of‐plane traction stress heatmaps (right) for EV (top panel) and RAB5A‐expressing (bottom panel) spheroids spreading on 15 kPa soft PDMS substrates. Scale bar, 50 µm. e) Radial profiles over time of in‐plane (left) and out‐of‐plane (right) traction stresses for representative examples of EV (top) and RAB5A‐expressing (bottom) spheroids wetting on 15 kPa soft PDMS substrates. Curves were smoothed using Lowess models on MATLAB. f) Period between maxima of in‐plane traction stress in radial profiles. Data are the mean with SEM, *n* = 3 independent experiments. ^****^p < 0.0001, Mann‐Whitney test. g) Out‐of‐plane tractions at coordinates located between 0 and 50 µm from the center of a spheroid. Data are the mean with SEM, *n* = 3 independent experiments. ^****^p < 0.0001, *ns* not significant, Kruskal‐Wallis test.

Cells at the invading edge display specific mechanical properties in order to drive collective movement.^[^ ^49^ ^]^ A key downstream effector of RAB5A expression is the WAVE2 actin nucleator,^[^ ^8^ ^]^ which has been shown to drive the formation of highly dynamic protrusions at the migrating front of single cells. In our system, we observed the establishment of a convex, collective supracellular actin cable upon RAB5A induction, which persisted for several hours (**Figure** **4**a). In contrast, control spheroids were unable to maintain such a supracellular actin bundle at the leading edge, resulting in the disassembly of structures to the single‐cell level (Movie S4.1, Supporting Information). To assess the mechanical properties of this cohesive actin bundle, a collective feature of the migrating front, we employed atomic force microscopy (AFM) nanomechanical mapping coupled to Airy scan confocal imaging of actin cytoskeleton. We observed that the peripheral actin regions are enriched with thicker, more aligned and parallel actin structures upon RAB5A expression, as compared to the control condition (Figure 4b). In addition, the actin‐rich leading edge of RAB5A‐expressing spheroids displayed a higher average Young's modulus than for EV as measured by AFM (Figure 4b,c). Thus, RAB5A promotes the formation of a stiffer, more coordinated actin cable at the migrating front, indicating increased contractility and tension during 2D wetting. Sustained actin‐rich protrusions at the front also reflect rapid focal adhesion turnover, supporting efficient long‐range force transmission. In this context, we next investigated the traction patterns at the front and at the core of the spreading aggregates.

We used 15kPa soft polydimethylsiloxane (PDMS) substrates decorated with fiducial markers at the surface to infer the tractions exerted by the spheroids during the wetting.^[^ ^50^ ^]^ Besides the direction and extent of in‐plane traction components, we also computed the out‐of‐plane tractions to provide a more comprehensive understanding of the cell‐substrate interaction in this multicellular system (Movies S4.2 and S4.3, Supporting Information).^[^ ^51^ ^]^ The *z*‐direction profile of a traction vector is usually positive at the leading front, where the cell pulls upward on the substrate, but becomes negative toward the rear, where the cell pushes on the substrate to balance the total cell traction stress.^[^ ^52^ ^]^ Similarly, we could determine that control spheroids pulled the gel upward at the periphery, but exerted negative downward tractions under the core for an extended period of time (Figure 4d). This observation suggests that the spheroid core acts as a limiting element in wetting, exerting a compressive stress on the substrate as a consequence of the overall surface tension of the spheroid. In contrast, upon RAB5A expression, the spheroid core did not exert negative tractions, and even showed upward tractions at early stages of wetting (Figure 4d). Quantitatively, these mechanical changes in cell‐substrate interactions were best illustrated by differences in the radial profiles of traction stress from the center to the front of the spheroids. In control cell aggregates, the out‐of‐plane component was negative toward the center, where RAB5A‐expressing spheroids exerted slightly upward tractions instead (Figure 4e). As expected, the radial profiles of in‐plane tractions peaked at the leading front of the cell clusters for all timepoints studied (Figure 4e). Of note, we observed a decreasing magnitude and a higher velocity of the in‐plane traction stress peak over time at the periphery of RAB5A‐expressing spheroids, as the spheroid transforms into a planar cell sheet (Figure 4e,f). Finally, we quantified the extent of traction forces under the core of the spheroids at early timepoints, and confirmed that RAB5A‐expressing spheroids exert less compressive stress on the substrate (Figure 4g). Instead, control spheroids displayed a slight but significant increase in downward traction stress at early wetting timepoints, hereby supporting the solid behavior of the jammed core which resists the spreading (Figure 4g).

If jamming‐unjamming transitions serve as one physical gateway to tumor progression and invasion, the epithelial‐to‐mesenchymal transition (EMT) represents another biological, genetic program driving carcinoma dissemination. However, the extent to which unjamming and EMT, or partial EMT (pEMT), cooperate to facilitate tumor invasion remains poorly understood.^[^ ^53^ ^]^ Therefore, we aimed to investigate whether the wetting in DCIS spheroids exhibits indicators of (p)EMT.^[^ ^54^ ^]^ To explore this, we quantified the protein Slug, a known transcription factor that regulates the expression of genes involved in EMT in breast epithelium, including E‐cadherin, using Western blot analysis. Strikingly, we found a downregulation of Slug in RAB5A‐expressing monolayers during the cell streaming motility, even though this cellular system has enhanced invasion properties (Figure S2a, Supporting Information). This is consistent with previous observations indicating that, while RAB5A expression has no impact on total E‐cadherin levels, it promotes increased straightness of cell‐cell junctions and enhanced E‐cadherin turnover.^[^ ^10^, ^55^ ^]^

Next, we stained DCIS spheroids for vimentin, an intermediate filament known to serve as a marker for (p)EMT.^[^ ^56^ ^]^ Interestingly, we detected vimentin structures in both conditions, although they appeared more numerous in the control (not quantified, Figure S2b, Supporting Information). These structures were primarily located at the cell‐substrate interface, were mostly concentrated around the nuclei, highlighting vimentin's mechanoprotective role during instances of significant physical stress on the nuclei. The qualitative decrease in vimentin intensity observed upon RAB5A expression may support the previously noted reduction in Slug protein levels, reinforcing the idea that RAB5A does not primarily activate a mesenchymal genetic program. In this context, we hypothesize that actomyosin is the primary cytoskeletal structure involved during fluid wetting.

Thus, RAB5A expression promotes complete wetting by mechanical reprogramming of spheroid tractions, contractility, and active surface tension. To support this observation, we used blebbistatin to decrease spheroid contractility and scored increased *V*_RMS_ of cell nuclei in the control condition (Figure S3, Supporting Information). Conversely, blebbistatin treatment delayed the wetting of RAB5A‐expressing spheroids, with a decrease in cell speed and persistence length—possibly due to a perturbation of the known RAB5A‐mediated cell‐cell junctions dynamical tuning (Figure S3, Supporting Information).^[^ ^10^ ^]^ Nonetheless, the significant differences in ROP and *L*_pers_ between RAB5A‐expressing and control spheroids, even under blebbistatin treatment, suggest that enhanced wetting is not driven solely by increased force transmission, but likely results from the combined effects of integrin upregulation and actin cortex reorganization.

### 2.5 Fluid‐Like Wetting Is Accompanied by Softening of the Entire Spheroid

Tumor softening is a sign of malignancy and is associated with the early steps of the metastatic cascade.^[^ ^57^ ^]^ During this process, a glassy‐like tumor softens to enhance cell escape through the dense ECM, and migrate toward secondary sites. Additionally, the oncogenic activation of normal epithelial cells at the single‐cell level has been shown to promote softening.^[^ ^58^ ^]^ Consequently, we investigated whether changes in rigidity also influence the 3D‐to‐2D spreading of DCIS spheroids.

**Figure 5:**
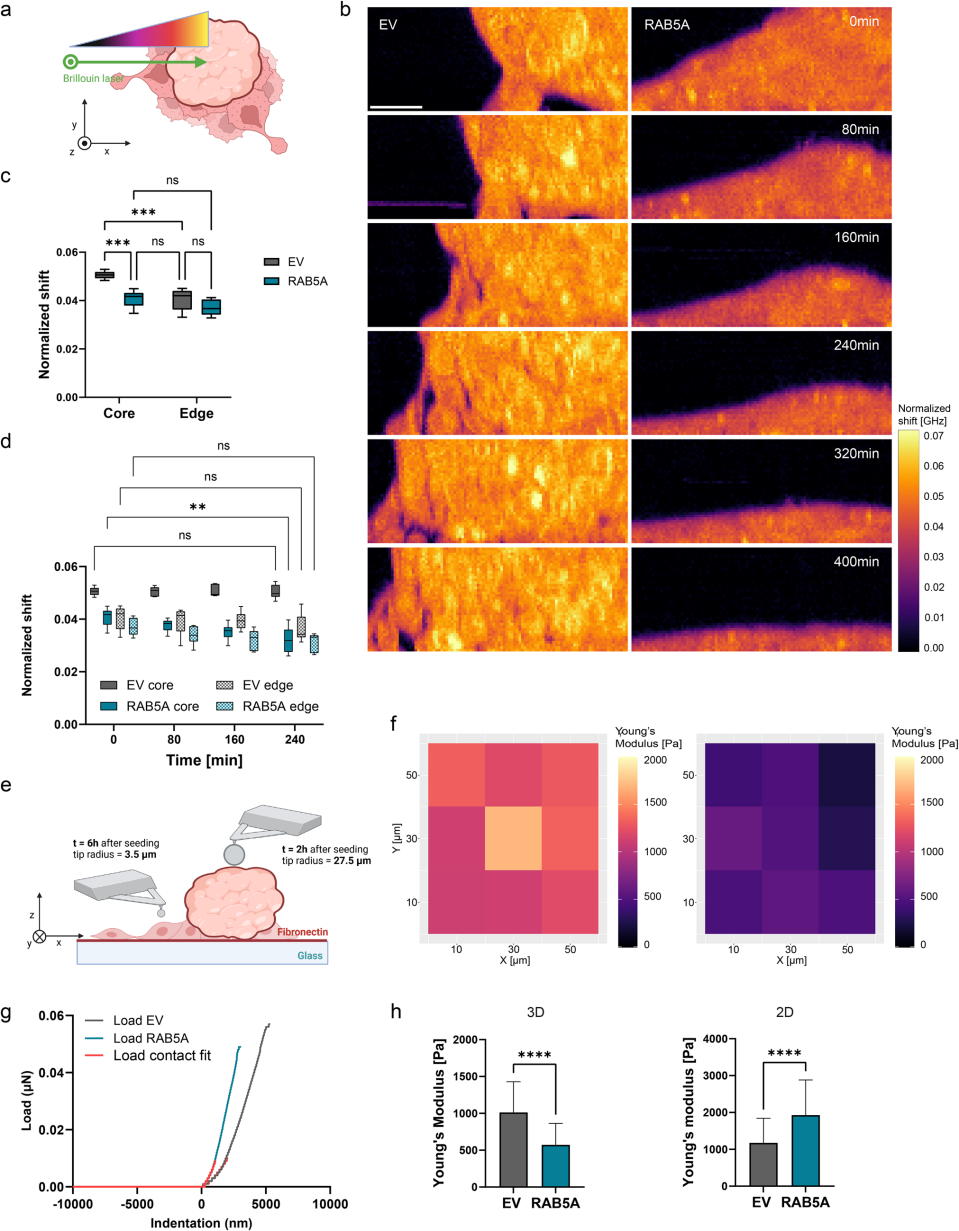
Softening drives spheroid 3D‐to‐2D solid‐to‐fluid transition. a) Scheme of Brillouin microscopy wetting experiment. Confocal slices are acquired in the ($\vec{r}$, $\vec{z}$) plane, where $\vec{r}$ is a position vector in the bottom adhesion plane, linear combination of $\vec{x},\;\vec{y},\;\vec{z}$ is the altitude vector. Image was generated using BioRender. b) Time‐lapse Brillouin shift images of EV (left), RAB5A‐expressing (right) spheroids wetting on a fibronectin‐coated glass substrate. Scale bar, 20 µm. c) Normalized Brillouin shift of the core and of the edge of spheroids at the onset of wetting. Data are min to max, *n* = 3 independent experiments. ^***^p < 0.001, *ns* not significant, two‐way ANOVA multiple comparisons. d) Normalized Brillouin shift of the core and the edge of spheroids over time. Data are min to max, *n* = 3 independent experiments. ^**^p < 0.01, *ns* not significant, two‐way ANOVA multiple comparisons. e) Scheme of nanoindentation experiment. Single cells were indented on top of their nuclei. Image was generated using BioRender. f) Representative indentation matrix scans (9 indentation points, 20 µm step) of representative examples of EV (left), RAB5A‐expressing (right) spheroids 2h after seeding on fibronectin‐coated glass substrates. g) Representative load‐indentation curves of representative examples of EV, RAB5A‐expressing single cells 6h after seeding on fibronectin‐coated glass substrates. Cells were indented at the center of the nucleus. h) Average Young's modulus of spheroids (left) and single cells (right) according to the configuration shown in e). Data are the mean with SD, *n* = 3 independent experiments. ^****^p < 0.0001, Mann‐Whitney test.

Stiffness measurements can often be limited as they typically provide either localized information or average the rigidity across an entire biological sample. To address these limitations, we employed Brillouin microscopy, which allows us to infer a proxy for the stiffness of the spheroids in a volumetric manner during the wetting (**Figure** **5**a).^[^ ^59^, ^60^ ^]^ By measuring the normalized Brillouin frequency shift *ν*_B_ ^*^ in the spheroid, which is directly proportional to the high‐frequency elastic longitudinal modulus (assuming that the term *ρ n* ^−2^ is constant, with *ρ* the density, *n* the refractive index of the sample),^[^ ^61^, ^62^ ^]^ we could define a volumetric map of stiffness over time. Strikingly, we observed that RAB5A expression robustly decreased the cell Brillouin shift compared to the control (Figure 5b). Furthermore, the shift values were homogeneous throughout the spheroid body upon RAB5A induction at the onset of wetting, when the edge and core regions in the EV condition exhibited sharp discrepancies (Figure 5c). The latter differences were reminiscent of the solid‐to‐gas type of transition previously observed for the control condition, where single motile cells tended to detach from the solid spheroid mass. Of note, we did not find significant differences between the Brillouin shift of the edge of the control condition and the edge or core of RAB5A‐expressing cell clusters (Figure 5c). These measurements indicate that the rigidity of the spheroid core might serve as a significant barrier to wetting, which can be alleviated by RAB5A expression. This assertion was further supported by the evolution of the normalized Brillouin shift over time, scoring a significant softening of the aggregate core upon RAB5A expression, but no strong change in longitudinal modulus for the other segmented areas, with or without RAB5A induction (Figure 5d).

Brillouin microscopy is based on the scattering of laser light by acoustic waves, and thus derives frequency shift values in the GHz range, which is several orders of magnitude higher than frequencies cells experience in vivo. To validate the previous results, we performed nanoindentation to infer a local average Young's modulus at the onset of wetting, and at later timepoints the stiffness of single cells belonging to the precursor film (Figure 5e).^[^ ^63^ ^]^ Consistent with the previous results, we scored a decrease in stiffness for the entire spheroid body upon RAB5A induction (Figure 5f,h). Interestingly, RAB5A‐expressing cells in the expanding monolayer displayed stiffer nuclei 6h after the wetting onset, as compared to the control (Figure 5g,h). This observation, in accordance with previous stiffness measurements in unjammed dense monolayers, highlights the mechanoprotective response of the nuclei undergoing large physical stresses during motility reawakening.^[^ ^10^, ^19^ ^]^ Thus, tissue fluidification shows stiffness plasticity during the wetting, as a soft multicellular cluster transforms into a planar sheet of stiffer cells.

The previous results show that the 3D‐to‐2D wetting is fostered by changes in cell mechanical properties. In that context, RAB5A promotes spheroid softening during complete wetting, but drives nucleus stiffening in 2D cell systems. These observations might support the fact that RAB5A expression gives multicellular systems an advantage to invade in multiple dimensions. Of note, the influence of matrix confinement on the invasion would give valuable insight on the crosstalk between cell‐cell strength, spheroid softening, and the generation of contractile forces in 3D.^[^ ^64^ ^]^

## 3 Discussion

In this work, we demonstrate that RAB5A significantly accelerates spheroid wetting by inducing a transition to a fluid‐like, highly collective type of motion. This behavior emerges from coordinated mechanical changes across scales, involving both cell‐substrate and cell‐cell interactions.

Upon RAB5A expression, spheroids undergo complete wetting, rapidly flattening into a monolayer with no apparent contact angle. In contrast, control spheroids reach a monolayer state only after a much longer timescale whileretaining a central cap‐like region during early spreading, consistent with the behavior of contractile droplets. Our data highlight key active mechanisms underlying this differential spreading, including enhanced focal adhesion turnover, formation of a supracellular actin cable, and reduced spheroid stiffness. Together, these results support a shift toward extensile active stresses upon RAB5A expression, thereby driving efficient aggregate flattening.

Functionally, RAB5A mediates endo‐, exocytic cycles of cargoes critical for cell invasion, and positively regulates specific integrin subtypes. This enhanced trafficking activity at the wetting front is directly coupled to increased FA kinetics and supracellular actin polarization, resulting in efficient force transmission at a collective scale. Interestingly, we found no evidence of increased fibronectin matrix deposition upon elevated level of RAB5A expression, indicating that increased integrin expression rather than augmented ECM deposition underpins the RAB5A‐induced wetting. However, the delayed wetting observed on laminin‐rich substrates could be related to the gradual ECM deposition.

In this work, we show that RAB5A promotes complete wetting not only by reprogramming the adhesion phenotype, but also by tuning mechanical properties at different scales. In particular, our results support the notion that tissue fluidification contributes to homogenize the local mechanical properties of a cellular ensemble. In line with this, we showed that RAB5A‐expressing spheroids exhibited decreased stiffness, with no significant difference between the center and the front of spreading, unlike the control. In addition, the core of the aggregate can flow upon RAB5A induction, whereas the control spheroids exert negative downward traction forces on the substrate under the jammed core for an extended period of time. Thus, RAB5A can mediate the escape from a jammed, solid‐like state which is represented by the core of control DCIS spheroids during wetting.

A significant implication of our research is that RAB5A modifies the mechanical properties of cell groups to promote collective cell migration. To investigate this, we measured the stiffness of the spheroids during the early stages of wetting, assessed the stiffness of individual nuclei within the wetting monolayer. Notably, we discovered that increasing the stiffness of individual nuclei through RAB5A expression leads to an overall softening of the entire cell assembly.

This apparent decoupling between nuclear and tissue‐scale mechanics raises intriguing questions about the interplay between intracellular stiffness and collective material behavior. While analogies with passive soft matter systems—such as dense suspensions of deformable particles—offer a useful conceptual framework,^[^ ^65^ ^]^ we recognize their limitations when applied to active, living systems. In our context, nuclei are not in direct contact, their deformation does not necessarily determine the packing or mechanical response of the entire aggregate. Therefore, we refrain from asserting a direct causal relationship. Future investigations combining high‐resolution nuclear deformation mapping and spatial analysis within 3D spheroids will be essential to determine whether RAB5A modulates nuclear organization in a way that contributes to tissue‐level unjamming, and subsequent mechanical fluidification.

Spheroid wetting is an active mechanism, based on a tug‐of‐war between contractility, traction, and surface tension.^[^ ^16^, ^17^ ^]^ In this framework, using Brillouin imaging, we observed that the spheroid core becomes softer upon RAB5A expression, indicating a more fluid‐like state. This suggests that in our system, cell‐cell communication within the spheroid is influenced not only by physical factors like steric repulsion, or nucleus deformation, but also by active processes such as cell‐cell adhesion mechanics, and cell movement. Moreover, previous studies have shown that RAB5A expression increases cell surface contacts and enhances local junctional tension.^[^ ^10^ ^]^ Therefore, further investigation into the dynamics of cell‐cell junctions within the spheroid is necessary to disentangle the contributions of cell‐cell adhesion strength and cell velocity to the overall stiffness of the spheroid.

We discussed in this study the effect of the elastic modulus, but did not fully characterize the viscosity of the spheroids during the wetting. Brillouin microscopy can give access to viscoelastic properties via the measurements of the frequency shift and of the spectral linewidth.^[^ ^59^, ^60^ ^]^ However, Brillouin spectroscopy infers a viscosity at very high frequencies (GHz), which correspond to short timescales for cell response. Although other viscosity measurements could bring insights on the flowing properties of the whole spheroids, as well as on energy dissipation in the system (frequency sweep or stress‐strain oscillation tests), small frequencies are needed to picture spheroid viscosity over a relevant timespan for wetting (0.1–1 mHz). Therefore, we can simply posit in our system that RAB5A‐expressing aggregates are less viscous than the control as they flow during the wetting.

We have identified several determinants of the solid‐to‐fluid transition during wetting. Nevertheless, the order in which they coordinate the spreading remains to be fully clarified. In terms of stiffness, RAB5A‐expressing spheroids exhibited a lower Brillouin shift at early timepoints of wetting as compared to the control condition. In addition, the altered tractions exerted by the spheroid core upon RAB5A induction suggest that mechanotransduction responses are likely at play. We posit that tumor softening together with increased cell‐substrate interaction, due to positive regulation of specific integrins, rule the initial steps of the wetting. At later stages, increased kinetics of FA assembly/disassembly supracellular actin cortex might, instead, primarily drive multicellular cluster polarization, (outward) radial migration, long‐range force transmission. In the active wetting framework, tissue tension might also impact the wetting dynamics.^[^ ^16^, ^17^, ^66^ ^]^ To verify this possibility, monolayer stress microscopy might have been employed to gain insights about the tension of the cell clusters at later timepoints. However, due to the longer time required by control spheroids to transform into planar cell sheet, this analysis could not be carried out for our system. Although not directly measured here, actin treadmilling has also been proposed as a driver of nonequilibrium transitions in active droplets, promoting the formation of protrusive layers, symmetric “fried‐egg” shapes with central aster‐like defects.^[^ ^67^ ^]^ Investigating whether RAB5A modulates actin treadmilling could further illuminate how cytoskeletal dynamics contribute to unjamming, shape remodeling during wetting.

## 4 Conclusion

Collective tumor invasion is a fundamental biological and physical component of cancer metastasis. A critical pathway to collective migration is the unjamming transition, wherein multicellular clusters shift from solid‐like to fluid‐like states through alterations in adhesion, motility, and density. In this study, we employed RAB5A expression as a model to induce tissue fluidification in DCIS spheroids and examined the mechanical characteristics of their wetting.

Our findings reveal significant alterations in both cell‐cell, cell‐substrate interactions upon RAB5A induction. Control spheroids exhibit a solid, contractile core that exerted pushing forces on the substrate, alongside a softer, invading edge. In contrast, fluidized aggregates demonstrate reduced and homogeneous stiffness throughout, gradual softening over time, with traction forces concentrated beneath the active core at early time points. During fluid wetting, migration velocity increases from the core to the leading front, where stiff, persistent supracellular actin bundles facilitate enhanced cell collectivity, tensional state in 2D, and long‐range force transmission.

At the molecular level, RAB5A perturbation disrupts the endo‐ and exocytic cycles of cargo essential for cell migration, promotes the assembly and disassembly dynamics of adhesion complexes, and upregulates specific integrin heterodimers. Consequently, RAB5A expression orchestrates a coordinated mechanical framework between the front and rear of multicellular clusters, driving an active, fluid‐like wetting. Notably, the regulation of adhesion mechanics by elevated RAB5A levels is pivotal for understanding coordinated migration in more complex multicellular systems.

## 5 Experimental Section

### Cell Lines, Culture Conditions

MCF10.DCIS.com cells were a gift from John F. Marshall (Barts Cancer Institute, Queen Mary University of London, UK), were cultured in Dulbecco's Modified Eagle Medium: Nutrient Mixture F‐12 (DMEM/F‐12, Biowest) supplemented with 5% horse serum (New Zealand Origin, Thermo Fisher Scientific), 0.5 µg mL^−1^ hydrocortisone (Sigma‐Aldrich), 10 µg mL^−1^ insulin (Millipore Sigma), 20 ng mL^−1^ epidermal growth factor (EGF) (Peprotech), 1% L‐glutamine (Thermo Fisher Scientific), 1% penicillin/streptomycin (Thermo Fisher Scientific). Cells were cultured in 100‐mm cell culture‐treated dishes (Corning), maintained at 37 °C, 5% CO_2_ in humidified atmosphere.

MCF10.DCIS.com cells were infected with pSLIK‐neo‐EV (empty vector control) or pSLIK‐zeo‐HA‐RAB5A lentiviruses generated by Gateway Technology (Invitrogen), selected with neomycin or zeocin to obtain stable doxycycline‐inducible cell lines. pBABE‐puro‐H2B‐GFP or pBABE‐puro‐H2B‐mCherry plasmids were provided by the istituto fondazione di oncologia molecolare (IFOM) Imaging Facility, used for retroviral infection to achieve constitutive expression of H2B‐GFP or H2B‐mCherry.

GFP‐Paxillin‐overexpressing cells were generated using lentiviral transduction. Third generation lentiviral particles were produced in HEK‐293FT cells (provided by the Genome Editing Core at Turku Bioscience Centre) using the pLenti6.3‐Paxillin‐EGFP (enhanced green fluorescent protein) plasmid (Addgene #187688). DCIS‐Rab5‐H2B‐mCherry cells were plated on a 10‐cm dish, transduced with fresh supernatant containing lentivirus particles, in the presence of Lipofectamine 2000 to aid transduction. Media were changed after 24 h, GFP‐positive cells were selected using the Sony SH800 Cell Sorter one week after transduction.

Cells were harvested every 2–3 d by washing each dish with 5 mL dulbecco's Phosphate‐buffered saline (DPBS), then incubating with 2 mL of 0.05% Trypsin at 37°C, 5% CO_2_. Trypsin digestion was stopped by addition of 5 mL growth medium, cells were centrifuged at 800 rpm for 5 min. After the supernatant was discarded, the pellet was resuspended in complete growth medium. Cells were usually split every 48–72 h in 1:6 or 1:10 ratio.

### Streaming Assay

Cells were seeded in culture‐treated six‐well plates (1.5 × 10^6^ cells per well) in complete culture medium and grown for 24 h to form a confluent monolayer. 16h prior to the experiment, RAB5A expression was induced by adding 2.5 µg mL^−1^ doxycycline (Sigma‐Aldrich) in fresh complete medium. At the start of observation, fresh media containing doxycycline was added again, and images were acquired every 10 min over a 20h period using ZEISS Axiovert Z1 microscope with a 10 × objective. During imaging, the cells were maintained at 37 °C, 5% CO_2_ in an environmental microscope incubator. For each sample, several independent field of views (FOVs) around the center of the well were captured in phase contrast and epifluorescence modes.

### Cell Lysis, Immunoblotting

Six hours after the second doxycycline induction, cells from hyperconfluent monolayers were washed with cold phosphate buffered saline (PBS), and collected in TX lysis buffer (TXLB; 50 mm Tris‐HCl, pH 7.5, 150 mm NaCl, 0.5% Triton‐X, 0.5% glycerol, 1% sodium dodecyl sulfate (SDS), complete protease inhibitor (Sigma‐Aldrich, 5056489001), phosstop tablet (Sigma‐Aldrich, 4906837001). Equal amounts of protein were loaded and resolved by SDS–polyacrylamide gel electrophoresis (4–20% MiniPROTEAN TGX gels, Bio‐Rad, 456–1096). Proteins were transferred to a nitrocellulose membrane (Bio‐Rad, 1704159) using the Trans‐Blot Turbo transfer system (Bio‐Rad) and membrane were blocked with StartingBlock blocking buffer (Thermo Fisher Scientific, 37538) for 1h at room temperature. Membranes were incubated with primary antibodies (monoclonal mouse anti‐RAB5A, #46449, Cell Signaling Technology, dilution 1:1000; monoclonal rabbit anti‐ITGAV (integrin alpha V), #ab179475, Abcam, dilution 1:500; polyclonal rabbit anti‐ITGA2, #AB1936, Chemicon, dilution 1:1000; monoclonal mouse anti‐ITGB1, #610468, BD Biosciences, dilution 1:1000; polyclonal rabbit anti‐ITGA5, #PA5‐82027, Invitrogen, dilution 1:1000; monoclonal rabbit anti‐ITGB5, #3629, Cell Signaling Technologies, dilution 1:1000; monoclonal rat anti‐ITGa11, #MAB4235, R&D systems, dilution 1:1000; monoclonal rabbit anti‐ITGB3, #ab179473, Abcam, dilution 1:200; polyclonal rabbit anti‐ITGA3, #ab131055, Abcam, dilution 1:1000; polyclonal rabbit anti‐ITGA6, #ab97760, Abcam, dilution 1:1000; monoclonal mouse anti‐ITGB4, #MAB1964, Merck Millipore, dilution 1:1000; polyclonal rabbit antifibronectin, #F3648, Sigma‐Aldrich, dilution 1:1000; monoclonal rabbit antislug (C19G7), #9585S, dilution 1:1000; monoclonal rat anti‐HSC70/HSP73 (1B5), #ADI‐SPA‐815, Enzo, dilution 1:1000; monoclonal rabbit anti‐Histone H3 (D1H2), #4499S, Cell Signaling, dilution 1:1000; monoclonal mouse antiglyceraldehyde‐3‐phosphate dehydrogenase (GAPDH), #5G4MaB6C5, HyTest, dilution 1:2000) in blocking buffer, followed by incubation with fluorophore‐conjugated secondary antibodies (AzureSpectra AC2165, AC2166, AC2135, AC2169, AH Diagnostics) for 30 min at room temperature. Membranes were scanned with the Azure Sapphire RGBNIR imaging system (AH Diagnostics). Integrin expression was quantified using Image Studio Lite v.5.2, normalized using housekeeping proteins HSP73, Histone3 or GAPDH. Quantifications were performed on the raw data obtained straight from the scanner. Results from 3 independent experiments were plotted, and their statistical significance analyzed using GraphPad Prism.

### Spheroid Formation

Cancer spheroids were generated using 24‐well AggreWell 400 plates (STEMCELL Technologies) following the manufacturer's protocol.^[^ ^68^ ^]^ Within each well there are 1200 pyramid‐shaped microwells with 400 µm side length, which allow the formation of monodisperse spheroids from distinct numbers of cells. In brief, 500 µL of antiadherence rinsing solution (STEMCELL Technologies) was added to each well, and the plate was centrifuged at 2000 × *g* for 2 min to remove air bubbles. After incubating at room temperature (RT) for at least 30 min, the rinsing solution was aspirated, and the plate was washed thrice with PBS. Subsequently, 400 µL of prewarmed medium with 40 ng mL^−1^ EGF was added to the wells, and the plate was centrifuged again at 2000 × *g* for 2 min. Next, cells of the desired cell lines were counted using a Neubauer chamber or a Countess Automated Cell Counter (Invitrogen Thermo Fisher Scientific), and suspensions of a precise number of cells (usually 300 000 to achieve 250 cells per spheroid at seeding) in 400 µL of medium (without EGF) were added to each well. The plate was then centrifuged at 200 × *g* for 5 min and the spheroids were grown at 37°C, 5% CO_2_ under humidified atmosphere overnight.

16h before performing an experiment, 400 µL of medium in each well were carefully replaced with fresh medium supplemented with 40 ng mL^−1^ EGF and 5 µg mL^−1^ doxycycline. For harvesting, the medium was collected from each well, centrifuged at 1500 × rpm for 5 s, before the spheroids were resuspended in complete medium containing 2.5 µg mL^−1^ doxycycline for further use. Where indicated, SPY650‐FastAct staining (Spirochrome) was incubated with the spheroids for 2h (1/1500 dilution from reconstituted stock following the manufacturer's protocol), and added in the medium for all imaging time.

### Wetting Assay

Substrates made of glass coverslip [# 1 (Carl Roth/Epredia), or # 1.5H (Carl Roth)], or soft PDMS (DOWSIL CY 52–276) were coated with 7 µg mL^−1^ fibronectin bovine plasma (Sigma‐Aldrich), Collagen‐I (Corning), or laminin from EHS murine (Sigma‐Aldrich) in DPBS (w/o magnesium, w/o calcium) from 1h at RT to overnight at 4 °C. Surfaces were then washed thrice with DPBS, and harvested spheroids were seeded usually at a concentration of 600 aggregates per 35 mm well. The spheroids were left for equilibrium at 37 °C, 5% CO_2_ for at least 30 min, and subsequently imaged for time‐lapse microscopy with NIKON Ti2 or ZEISS Axiovert Z1 microscopes unless otherwise specified.

### PIV Analysis, Collective Metrics

For a given experiment, time‐lapse images from different spheroids for each condition were simultaneously collected. Nuclei images were recorded at the cell plane directly in contact with the bottom substrate. Time‐lapse fluorescent images of cell nuclei were analyzed using a custom PIV algorithm coded in MATLAB (Math Works Inc.), from an initial script written by Dr Ong Hui Ting mechanobiology institute (MBI, Singapore). Interrogation window was set at 64 × 64 pixels, with an overlap of 50% between adjacent windows.

The instantaneous *V*_RMS_(*t*) of the nuclei within one spheroid, i.e., one FOV, at a timepoint *t* was computed as

(1) $$V_{\mathrm{RMS}}\left(t\right)=\sqrt{{\langle {\left|v\left(t\right)\right|}^{2}\rangle }_{x}}$$

where *v*(*t*) is the instantaneous velocity vector, and 〈.〉_*x*_ indicates an average over grid points *x* corresponding to the centers of the PIV interrogation windows with nonzero velocity vectors.

Mean squared displacements (MSD) of the nuclei were calculated as

(2) $$\mathrm{MSD}\left(\Delta t\right)={\left|r_{m}\left(t+\Delta t\right)-\;r_{m}\right|}^{2}$$

where *r_m_* (*t*) are the nuclei trajectories calculated by numerical integration of the instantaneous velocity fields as obtained from the PIV analysis. The average was performed over all the trajectories within a spheroid in the time window comprised between 1 and 12h after the start of the microscopy. To estimate the persistence length *L*_pers_ of the nuclear motion the MSD curves were fitted with a function of the form

(3) $$g\left(\Delta t\right)={\left(u_{0}\Delta t\right)}^{2}{\left[1+\left(\frac{u_{0}\Delta t}{L_{\mathrm{pers}}}\right)\right]}^{-1}$$

This expression describes the transition between a short‐time ballistic‐like scaling, with characteristic speed *u*_0_, and a long‐time diffusive scaling. The transition between the two regimes takes place for Δ*t ≈ u*_0_^−1^ *L*_pers_^−1^, that is, after the nucleus has traveled with an approximately constant velocity over a distance *L*_pers_.

An ROP to quantify isotropy of nuclei movement was computed as followed

(4) $$\mathrm{ROP}\left(t\right)=\frac{1}{2}{\langle \frac{z\left(t\right).v\left(t\right)}{\left|z\left(t\right)\right|\left|v\left(t\right)\right|}+1\rangle }_{x}$$

where *v*(*t*) is the instantaneous velocity vector at a given grid point, *z*(*t*) is the position vector from the center of the spheroid mass to the same grid point, 〈.〉_*x*_ indicates an average over grid points *x* corresponding to the centers of the PIV interrogation windows with nonzero velocity vectors. For instance, a value of ROP(*t*) *=* 1 corresponds to a perfectly outward‐directed movement from all cell nuclei from the FOV at time *t*.

### Immunostaining

Spheroids were fixed by washing the well once with prewarmed DPBS followed by 15 min incubation in prewarmed 4% paraformaldehyde (PFA) solution at 37 °C. Spheroids were gently washed three times with DPBS for 5 min after fixation. Fixed spheroids were stored in DPBS at 4 °C before staining.

For staining against first antibody, a blocking solution with 0.1% TritonX‐100, 1% bovine serum albumin (BSA) in DPBS was prepared, and added to previously fixed spheroids for 10 min. First antibody was diluted in blocking buffer, incubated 2h at RT or overnight at 4 °C. Spheroids were then washed twice with DPBS for 10 min. A third wash was performed in blocking buffer. Secondary antibody was diluted in blocking buffer, and incubated for 1h at RT in the dark. Spheroids were subsequently washed thrice with DPBS. 4',6‐diamidino‐2‐phenylindole (DAPI) was then optionally added at a concentration of 0.5 µg mL^−1^ and incubated for 30 min. Spheroids were rinsed twice with DPBS andmounted with MOWIOL.

The primary antibodies used for immunostaining were the following: monoclonal mouse anti‐RAB5A, #46449, Cell Signaling Technology, dilution 1:200; monoclonal rabbit antivimentin, #5741, Cell Signaling Technologies, dilution 1:100; polyclonal rabbit anti‐phosphopaxillin (Tyr 118), #2541, Cell Signaling Technology, dilution 1:200.

### Membrane Staining

Doxycycline‐treated DCIS spheroids (prepared as described above) were seeded on fibronectin‐coated glass substrates (MatTek) and left to attach for 45 min. Spheroids were then stained with wheat germ agglutinin (WGA, ThermoFisher Scientific) conjugated to Alexa Fluor 647 dye for 10 min at 37°, 5% CO_2_, washed once with fresh complete medium (without WGA dye), and subsequently imaged using a Nikon microscope equipped with confocal scanner unit W1 (CSU‐W1) Yokogawa Spinning Disk unit with a 40 × NIKON Plan‐Apochromat air objective numerical aperture (NA = 0.95, working distance (WD) = 250 µm). Several samples of EV, RAB5A‐expressing spheroids were prepared and stained every 2h to avoid membrane dye internalization by the cells.

### Focal Adhesions Imaging And Analysis

MCF10.DCIS HA‐RAB5A H2B‐mCherry GFP‐paxillin cells were harvested as spheroids as described previously. 16h before an experiment, RAB5A was induced in half the wells by addition of doxycycline. The other half of wells for control spheroids did not receive doxycycline treatment for this type of experiment. Spheroids were seeded in fibronectin‐coated 8‐well μ‐Slide (IBIDI) for imaging. Focal adhesion dynamics were recorded between 2 and 10h after spheroid seeding for 20 min every 30 s using a Zeiss microscope equipped with 3i CSU‐W1 Yokogawa Spinning Disk unit with a 63 × Zeiss Plan‐Apochromat Oil objective (NA = 1.4, WD = 190 µm) and environmental control (37 °C, 5% CO_2_). Focal adhesion dynamics were analyzed using the focal adhesion analysis webserver hosted from Shawn Gomez's Lab at the University of North Carolina at Chapel Hill.^[^ ^40^ ^]^ In brief, background subtraction (rolling ball 50 pixels) was applied to the time‐lapse images before loading them on the server. Threshold was set at 4, and minimal adhesion size was set at 3 pixels for all data analyzed. Rates of focal adhesion assembly and disassembly were further analyzed on GraphPad Prism.

Phosphopaxillin complexes were analyzed using the Analyze Particles plugin available from Fiji. In brief, a manual threshold was set to remove background staining signal, watershed correction was applied, and phosphopaxillin complexes with a cross‐sectional area of more than 0.15 µm^2^ were analyzed.

### Nanopatterning

Nanopatterned surfaces preparation was based on the self‐assembly of diblock copolymer micelles.^[^ ^69^ ^]^ In brief, coverslips were cleaned from organic contaminations, activated by Caro acid treatment (H_2_SO_5_) for at least 45 min, then washed with MilliQ water and subsequently sonicated for 5 min. Then, micelles of copolymers containing gold nanoparticles were spin coated on the coverslips to achieve a desired particle spacing. Polymers were removed from the surface by hydrogen gas plasma treatment (0.4 mbar for 45 min), and the substrates were passivated with PEG molecules (molecular weight 2000 g mol^−1^) to prevent cell adhesion and protein adsorption (incubation from overnight to 48h at 80 °C). Finally, surfaces were incubated with cyclic RGD peptides (25 µm) for 2h at room temperature, washed with MilliQ water, and directly used for wetting experiment.

### Traction Force Microscopy

Traction force microscopy (TFM) substrates were prepared using PDMS elastomer.^[^ ^50^ ^]^ Briefly, a 15 kPa soft PDMS mixture was prepared by mixing DOWSIL CY 52–276 A, B components in 1:1 ratio w/w. The mixture was spin coated onto glass coverslips (spin acceleration 250 rpm s^−1^, 500 rpm for 10 s, then 750 rpm for 40 s) and polymerized overnight at 70 °C. PDMS layers were then activated with a 1:10 (v/v) solution of 3‐aminopropyltriethoxysilane (Sigma‐Aldrich) diluted in 100% lab‐grade ethanol for 10 min at RT, washed thrice with 100% ethanol, and dried at 70 °C for 10 min. Substrates were incubated for 10 min at RT with a 2:1000 v/v solution of fluorescent beads (FluoSpheres carboxylate‐modified microspheres, 0.2 µm, Thermo Fisher Scientific) in MilliQ water previously sonicated for 10 min. Substrates were then washed thrice with MilliQ water and dried at 70 °C for 10 min. TFM surfaces were kept at RT in the dark no longer than 2 weeks.

Soft PDMS substrates were coated with fibronectin and spheroids were seeded the same way as described above. Z‐stacks of fluorescent bead images (slice interval of 0.6 or 0.2 µm) were acquired every 10 min using a Nikon microscope equipped with CSU‐W1 Yokogawa Spinning Disk unit with a 40 × NIKON Plan‐Apochromat air objective (NA = 0.95, WD = 250 µm) and environment control (37 °C, 5% CO_2_). A reference relaxed *z*‐stack fluorescence image of the beads was later acquired after removal of the spheroids with SDS.

The 3D traction field was computed as previously described.^[^ ^70^ ^]^ The 3D displacement field of the top surface of the gel was measured by comparing the Z‐stack images of the reference and deformed beads configurations using a custom PIV written in MATLAB.^[^ ^71^, ^72^ ^]^ Finally, the traction field was calculated, from the measured displacement field, by solving the elastostatic equation for a finite thickness gel in Fourier space.^[^ ^51^ ^]^

### Brillouin Microscopy

Glass coverslips (MATTEK P35G‐0‐20‐C) were coated with fibronectin and spheroids were seeded the same way as described before. The Brillouin microscope consisted of a commercial ZEISS body (Axiovert 200 M) coupled with a home‐built spectrometer based on a two‐stage VIPA configuration,^[^ ^73^ ^]^ with the addition of a Lyot stop to increase the suppression of the elastically scattered light.^[^ ^74^ ^]^ The excitation laser had a wavelength of 660 nm (Torus 660, NOVANTA PHOTONICS), and laser power and exposure were set at less than 6 mW on the sample plane, and 100 ms, respectively. A 488 nm and 560 nm laser coaligned with the Brillouin excitation were used for fluorescence imaging. Samples were imaged every 80 min in the ($\vec{r}$, $\vec{z}$) plane (40 µm × 100 µm window, with 1 µm resolution) with a ZEISS Plan‐Apochromat 40 × oil objective (NA = 1.00, WD = 0.17 mm), maintained at 37 °C, 5% CO_2_ in an environmental incubation chamber.

To compare the measured Brillouin frequency shift *ν*_B_ (BFS) between different conditions, a normalized BFS *ν*_B_ ^*^ was computed in respect to the BFS of the surrounding medium

(5) $$\nu_{B}^{*}=\frac{\nu_{B}-\nu_{B\;\mathrm{Medium}}}{\nu_{B\;\mathrm{Medium}}}$$

### Nanoindentation

To measure the stiffness of the spheroids, single cells microindentation measurements were performed using a PAVONE nanoindentation platform (Optics11 Life) according to the previously published methods.^[^ ^75^ ^]^ The measurements of the spheroids were carried out using a cantilever with 0.032 N m^−1^ stiffness and a 27.5 µm tip radius, while for the single cells a cantilever with 0.021 N m^−1^ stiffness and a 3.5 µm tip radius was used. During the probe calibration and measurements, the biological samples were kept in a controlled cell culture environment with 5% CO_2_ and 37 °C. After the device was calibrated, indentation experiments for spheroids and single cells were carried out in load control at 0.05 µN and speed of 2 µm s^−1^ followed by a hold time of 1 s and subsequent retraction at 2 µm s^−1^ on each location. Single cells were indented at the center of their nuclei. Indentation experiments for spheroids were carried out as matrix scans of 3 × 3 measurements with step size of 20 µm for each location. Data analysis was done using the Data Viewer (V2.5.0) software supplied by the device manufacturer. The Young's modulus (*E*) from each load‐indentation curve was calculated with the Hertzian contact model using a constant indentation speed.^[^ ^63^ ^]^ The contact point for each curve was determined using the software's integrated contact fit: 80% of the maximum load for spheroids, 20% for single cells. The Hertz fit was applied to a maximum depth of 2 µm for spheroids, while for single cell measurements, it was applied to a maximum load of 0.01 µN.

### Atomic Force Microscopy

Spheroids were seeded on fibronectin (7.5 µg mL^−1^)‐coated 35 mm glass bottom dishes (FluoroDish FD35‐100; World Precision Instruments) for 6h prior to imaging. While spreading, the cells were treated with SPY650‐FastAct (Spirochrome) to visualize the actin cytoskeletal structure. After locating spreading spheroids by transmitted light, AFM force nanomechanical mapping was performed at the invasive front. AFM data was collected using a JPK NanoWizard 4XP BioScience AFM system (Bruker) using Quantitative Imaging nanomechanical mapping modality. PFQNM‐LC‐V2 live cell probes (Bruker) were used, thus applying enough force to generate deformations of ≈1 µm (applied forces ranged between 2–6 nN for most spheroids). AFM maps were conducted at a resolution of 128*128 pixels, over areas of 40*40 µm^2^ (depending on the size of available spreading cells), with a *Z* length of 3 µm, Z speed of 150 µm s^−1^. To generate Young's modulus maps, a modified Hertz contact model for paraboloidal tips was used.^[^ ^76^ ^]^

During AFM mapping, a Z‐stack was taken for both fluorescent nuclei (488 nm or 587 nm) and actin (647 nm) channels, for both EV control and RAB5A conditions. Confocal images were taken using an LSM 900 microscope with Airyscan2 (Zeiss) using a 25x (0.8 NA, long distance live cell imaging (LD LCI) Plan‐Apochromat) objective lens (Zeiss) at the basal side of the spreading spheroid invasive front. Photomultiplier settings were identical for all conditions to allow direct comparison. Z‐stacks images were acquired with a step size of 500 nm over the bottom 5–10 µm of the invasive front to visualize the actin structures. Images in the figures are presented as maximum intensity Z‐projections, with Airy‐scan processing applied for image deconvolution.

### 3D Image Reconstruction

Z‐stack imaging data were denoised and reconstructed in volume using NIS Element software functions.

### Statistics

Statistical analysis was performed with GraphPad Prism 10 software. To compare two independent groups of data a nonparametric two‐sided *t*‐test (Mann‐Whitney test) was used. To compare more than two independent groups, a one‐way analysis of variance (ANOVA) with multiple comparison was performed. The significance between independent groups was determined using a two‐way ANOVA test with multiple comparisons. Asterixis corresponds to the following significance levels: ^*^ *p* ≤ 0.05, ^**^ *p* ≤ 0.01, ^***^ *p* ≤ 0.001, ^****^ *p* ≤ 0.0001. The number of independent experiments (*n*) considered for each plot is stated in the legend.

## Conflict of Interest

The authors declare no conflict of interest.

## Supporting information



Supporting Information

Supplemental Movie S1.1

Supplemental Movie S1.2

Supplemental Movie S1.3

Supplemental Movie S1.4

Supplemental Movie S2.1

Supplemental Movie S2.2

Supplemental Movie S2.3

Supplemental Movie S3.1

Supplemental Movie S3.3

Supplemental Movie S4.1

Supplemental Movie S4.2

Supplemental Movie S4.3

Supporting Information

Supporting Information

Supporting Information

## Data Availability

The data that support the findings of this study are available from the corresponding author upon reasonable request.
