# Supplementary material for: RAB5A Promotes Active Fluid Wetting by Reprogramming Breast Cancer Spheroid Mechanics
Source: Adv Sci (Weinh). 2025 Jul 25;12(34):e03569. doi: 10.1002/advs.202503569 (PMC12442610; doi:10.1002/advs.202503569)
Supplement: Supplementary file 1 — Supporting Information [file ADVS-12-e03569-s009.docx]

Supporting Information

RAB5A Promotes Active Fluid Wetting by Reprogramming Breast Cancer Spheroid Mechanics

*Grégoire Lemahieu, Paulina Moreno-Layseca, Tobias Hub, Carlo Bevilacqua, Manuel Gómez-González, Federica Pennarola, Federico Colombo, Andrew E. Massey, Leonardo Barzaghi, Andrea Palamidessi, Leon-Luca Homagk, Samuel F.H. Barnett, Alexander X. Cartagena-Rivera, Christine Selhuber-Unkel, Robert Prevedel, Xavier Trepat, Joachim P. Spatz, Johanna Ivaska, Giorgio Scita, Elisabetta Ada Cavalcanti-Adam^*^*

**
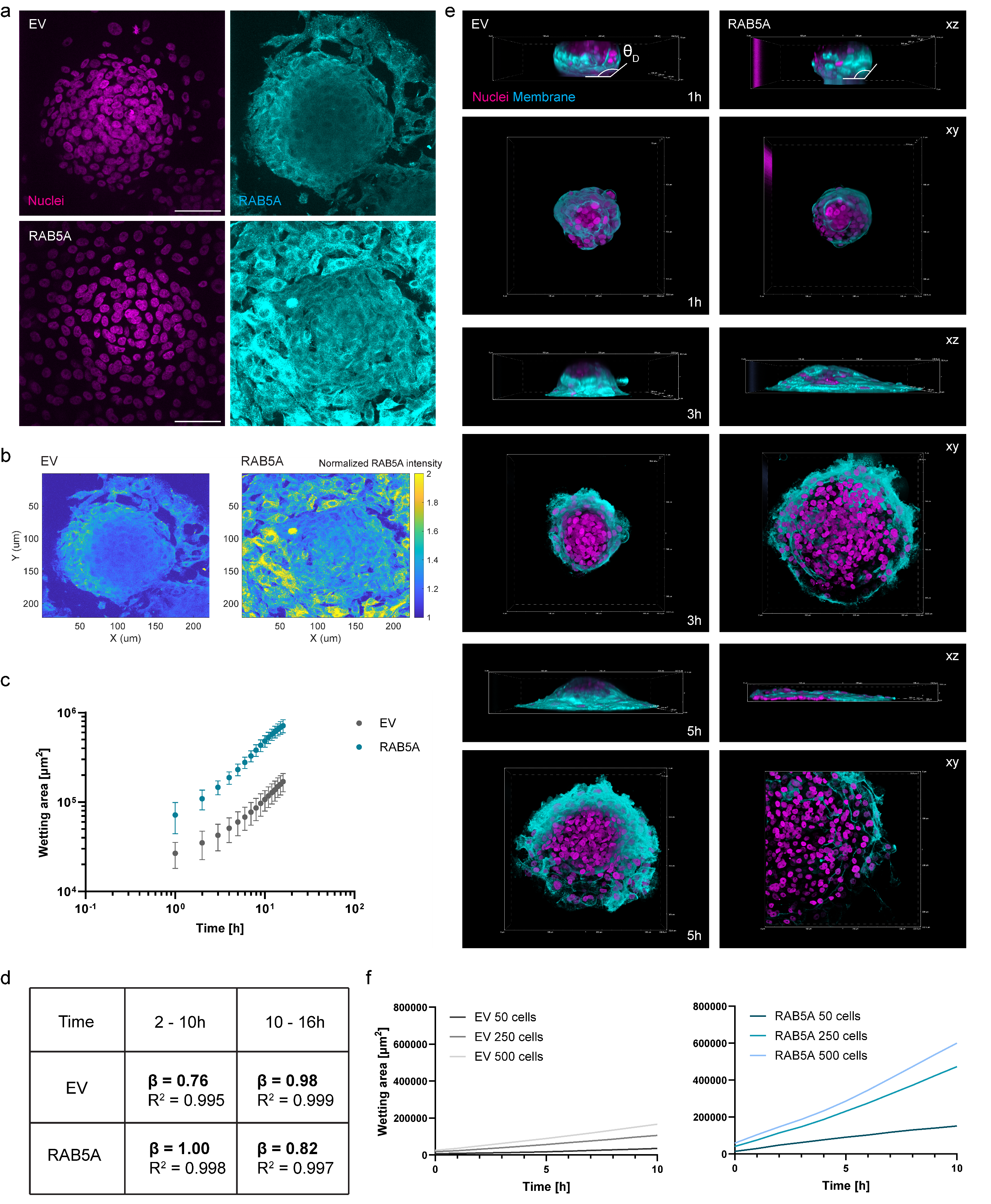
**

**Figure S1. RAB5A expression is associated with enhanced DCIS spheroid wetting**. a) Immunostaining images of RAB5A GTPase in representative examples of EV control (top) and RAB5A-expressing spheroids (bottom) spreading on fibronectin-coated glass substrate. Images were projected with maximal intensity over a height of 5 μm. Nuclei are in cyan, RAB5A in magenta. Scale bar, 50 μm. b) Heatmap of RAB5A signal intensity normalized to the background intensity for the examples shown in a. c) Evolution of the wetting area over time for EV and RAB5A-expressing spheroids. Data are the mean with SD, n = 3 independent experiments. d) Power law fit $S\left( t \right)=\alpha.t^{\beta}$, with *S* the spheroid wetting area, *t* the time, and *β* a fitted exponent. Increasing *β* between 0 and 1 indicates a solid-like to liquid-like type of transition. Fits are performed on the mean evolution of the wetting area over time for 15 different spheroids from 3 independent experiments. e) Representative 3D-reconstructed images of EV (left) and RAB5A-expressing (right) spheroids spreading on fibronectin-coated glass substrates. Side-view (xz) and bottom-view (xy) images; timepoints are indicated. Nuclei are in magenta; membrane is in cyan. f) Average wetting area of EV (left) and RAB5A-expressing (right) spheroids spreading on fibronectin-coated glass substrates as a function of the time and the cell seeding density per spheroid. n = 2 independent experiments.


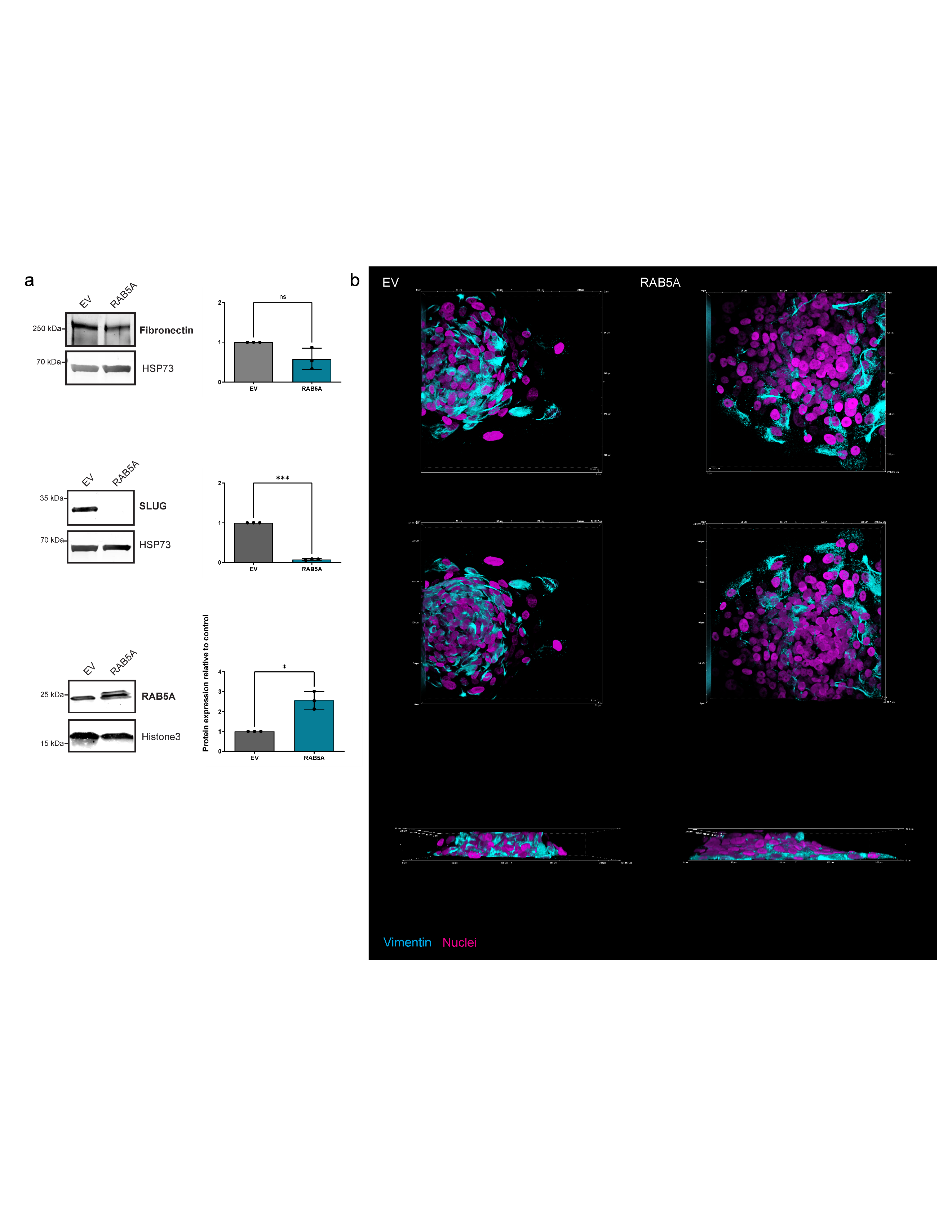


**Figure S2. RAB5A expression does not trigger a full mesenchymal invasive program**. a) Western blot quantification of Fibronectin, Slug and RAB5A proteins in control and RAB5A-expressing monolayers 6h after the second injection of doxycycline. Data are the mean with SD, n = 3 independent experiments. *^*^p* < 0.05, *^***^p* < 0.001, paired t test. b) Representative 3D-reconstructed images of vimentin filament staining in control (left) and RAB5A-expressing (right) spheroids wetting fibronectin-coated glass surface. Bottom (top), top (middle), and side (bottom) views. Spheroids were fixed 4h after seeding. Nuclei are in magenta; vimentin structures are in cyan.

**
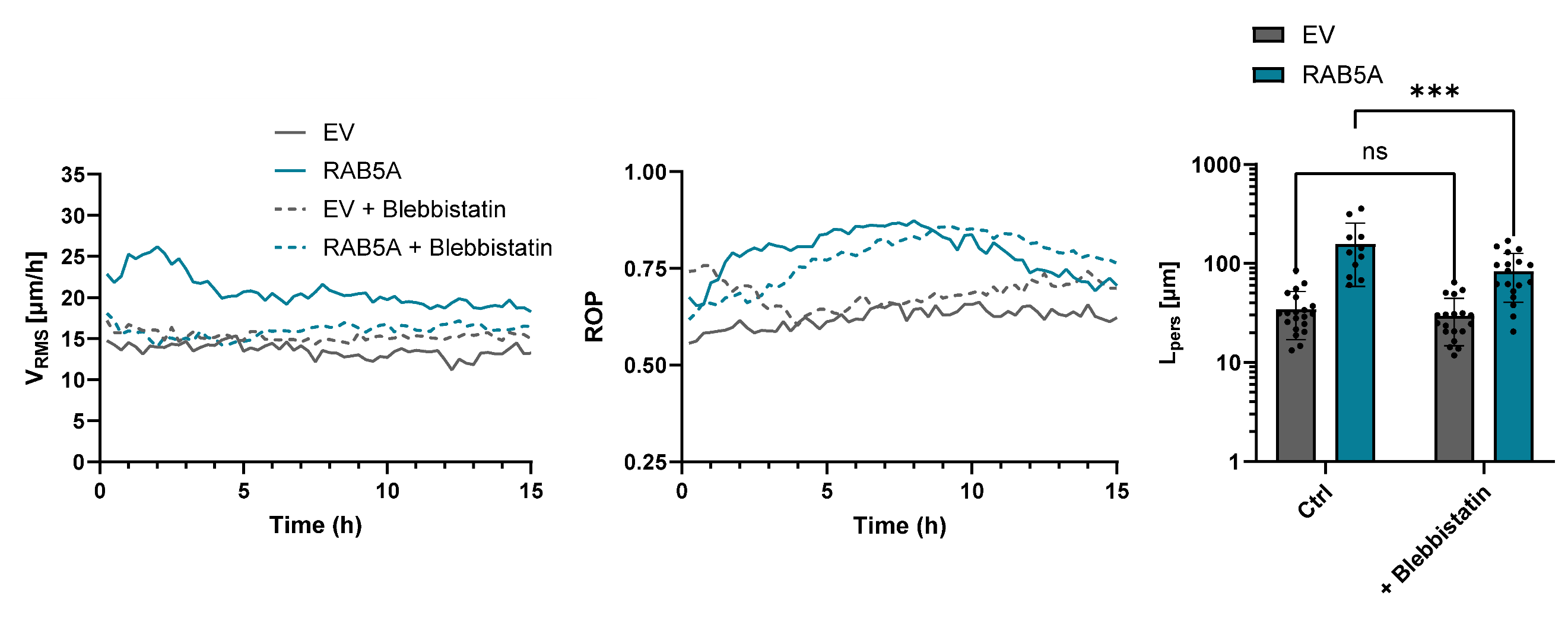
**

**Figure S3. Blebbistatin treatment impairs RAB5A-mediated fluid-like wetting.** *V_RMS_* (top), *ROP* (middle), and *L_pers_* (bottom) of control and RAB5A-expressing spheroids with and without addition of 30 μm blebbistatin. Persistence length was computed over 14h of migration. Data are the mean with SD, n = 2 independent experiments. *ns* not significant, *^***^p* < 0.001, 2-way ANOVA with multiple comparisons.

**Movie S1.1**

BF time-lapse of EV and RAB5A-expressing spheroids wetting on a fibronectin-coated glass substrate. Scale bar 100 μm.

**Movie S1.2**

Nuclei trajectories of EV and RAB5A-expressing spheroids wetting on a fibronectin-coated glass substrate. Scale bar 100 μm.

**Movie S1.3**

Bottom-view time-lapse 3D reconstruction of nuclei- and actin-labeled spheroids spreading on fibronectin-coated glass substrates.

**Movie S1.4**

Side-view time-lapse 3D reconstruction of nuclei- and actin-labeled spheroids spreading on fibronectin-coated glass substrates.

**Movie S2.1**

BF time-lapse of EV and RAB5A-expressing spheroids wetting on a fibronectin-coated glass substrate. Scale bar 100 μm.

**Movie S2.2**

BF time-lapse of EV and RAB5A-expressing spheroids wetting on a collagen-I-coated glass substrate. Scale bar 100 μm.

**Movie S2.3**

BF time-lapse of EV and RAB5A-expressing spheroids wetting on a laminin-rich glass substrate. Scale bar 100 μm.

**Movie S3.1**

Time-lapse of live GFP-paxillin in EV and RAB5A-expressing spheroids during wetting. Scale bar 25 μm.

**Movie S3.2**

BF time-lapse of EV and RAB5A-expressing spheroids wetting on nanopatterned substrates. Scale bar 100 μm.

**Movie S4.1**

Time-lapse imaging of FastAct in EV and RAB5A-expressing spheroids during wetting. Scale bar 50 μm.

**Movie S4.2**

Heatmaps of in-plane traction stresses for EV and RAB5A-expressing spheroids wetting on a 15-kPa soft PDMS substrate.

**Movie S4.3**

Heatmaps of out-of-plane traction stresses for EV and RAB5A-expressing spheroids wetting on a 15-kPa soft PDMS substrate.
