# Supplementary material for: RAB5A Promotes Active Fluid Wetting by Reprogramming Breast Cancer Spheroid Mechanics
Source: Adv Sci (Weinh). 2025 Jul 25;12(34):e03569. doi: 10.1002/advs.202503569 (PMC12442610; doi:10.1002/advs.202503569)

ADVANCED SCIENCE
Open Access

Supporting Information

for *Adv. Sci.*, DOI 10.1002/advs.202503569

RAB5A Promotes Active Fluid Wetting by Reprogramming Breast Cancer Spheroid Mechanics

*Grégoire Lemahieu, Paulina Moreno-Layseca, Tobias Hub, Carlo Bevilacqua, Manuel Gómez-González, Federica Pennarola, Federico Colombo, Andrew E. Massey, Leonardo Barzaghi, Andrea Palamidessi, Leon-Luca Homagk, Samuel F. H. Barnett, Alexander X. Cartagena-Rivera, Christine Selhuber-Unkel, Robert Prevedel, Xavier Trepat, Joachim P. Spatz, Johanna Ivaska, Giorgio Scita and Elisabetta Ada Cavalcanti-Adam**

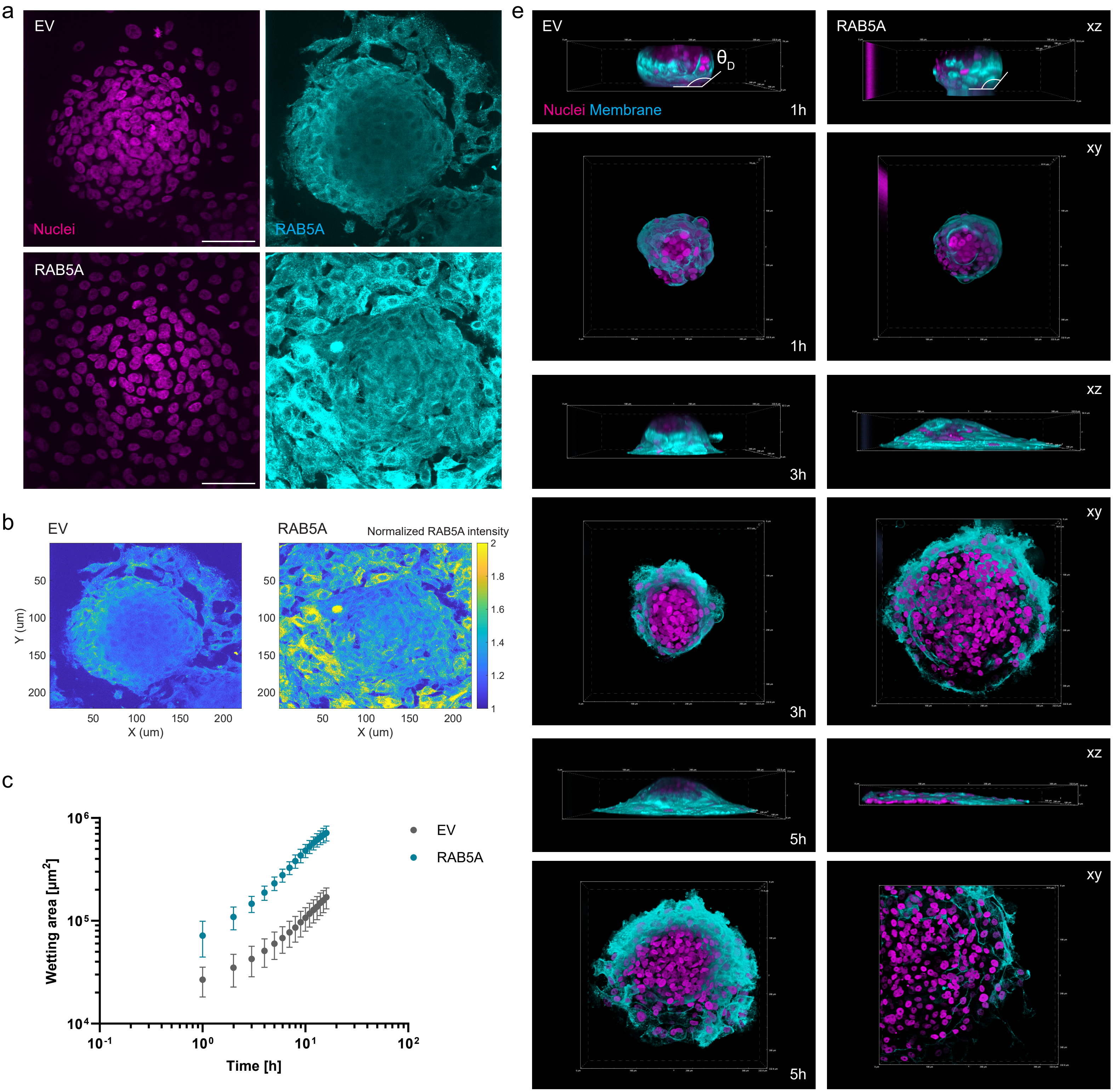

d

Time	2 - 10h	10 - 16h
EV	**β = 0.76** R^2 = 0.995	**β = 0.98** R^2 = 0.999
RAB5A	**β = 1.00** R^2 = 0.998	**β = 0.82** R^2 = 0.997

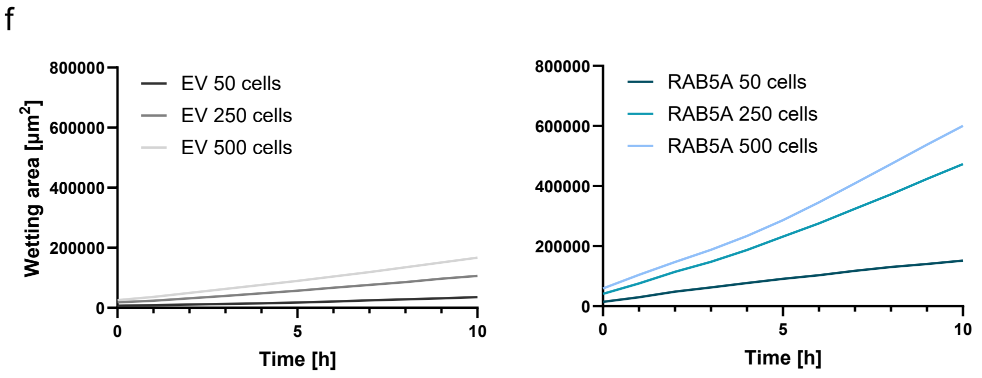

Supplement: Supplementary file 14 — Supporting Information [file ADVS-12-e03569-s008.pdf]
