# Supplementary material for: RAB5A Promotes Active Fluid Wetting by Reprogramming Breast Cancer Spheroid Mechanics
Source: Adv Sci (Weinh). 2025 Jul 25;12(34):e03569. doi: 10.1002/advs.202503569 (PMC12442610; doi:10.1002/advs.202503569)
Supplement: Supplementary file 15 — Supporting Information [file ADVS-12-e03569-s014.pdf]

ADVANCED SCIENCE
Open Access

Supporting Information

for *Adv. Sci.*, DOI 10.1002/advs.202503569

RAB5A Promotes Active Fluid Wetting by Reprogramming Breast Cancer Spheroid Mechanics

*Grégoire Lemahieu, Paulina Moreno-Layseca, Tobias Hub, Carlo Bevilacqua, Manuel Gómez-González, Federica Pennarola, Federico Colombo, Andrew E. Massey, Leonardo Barzaghi, Andrea Palamidessi, Leon-Luca Homagk, Samuel F. H. Barnett, Alexander X. Cartagena-Rivera, Christine Selhuber-Unkel, Robert Prevedel, Xavier Trepat, Joachim P. Spatz, Johanna Ivaska, Giorgio Scita and Elisabetta Ada Cavalcanti-Adam**

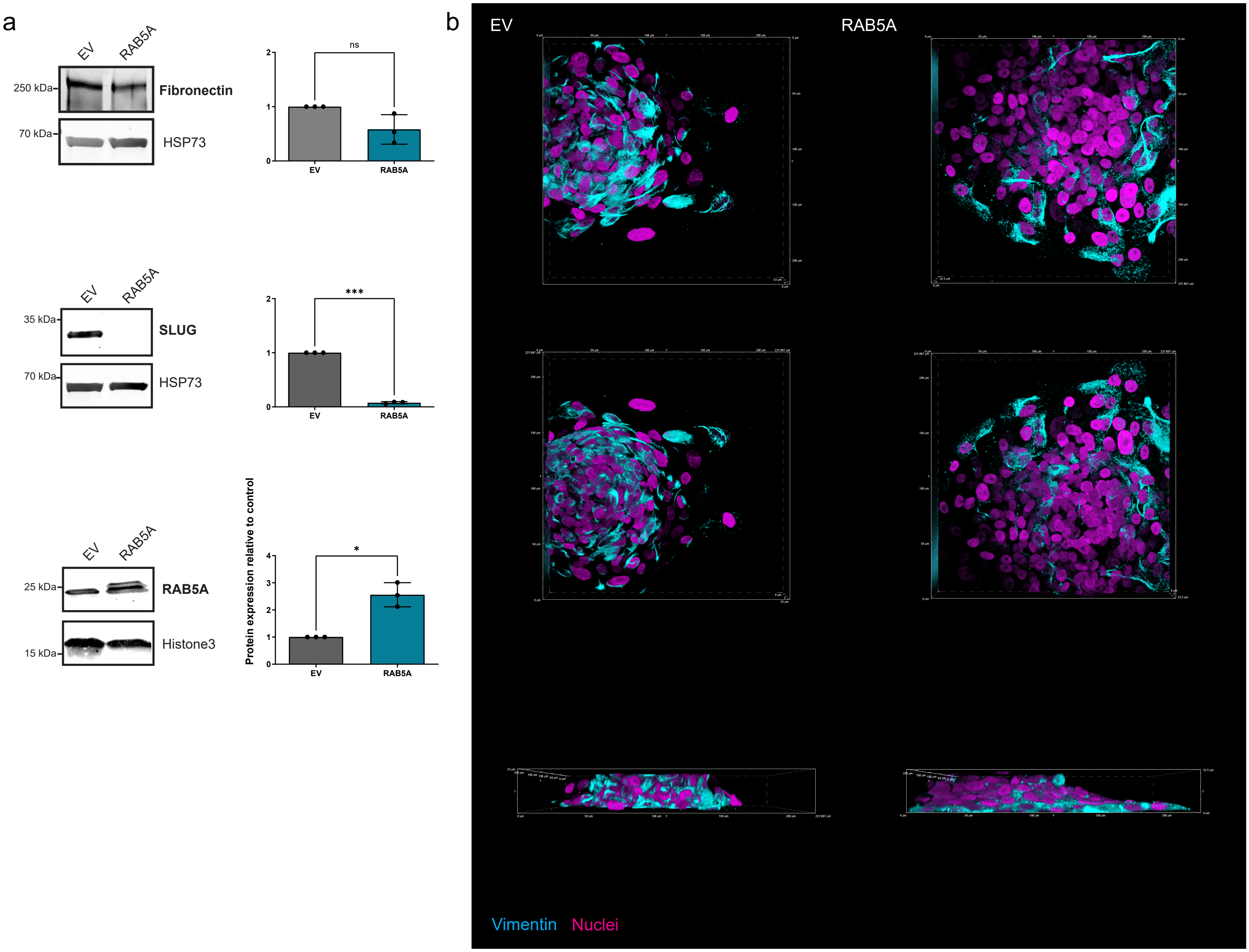
a
EV
RAB5A
250 kDa
Fibronectin
70 kDa
HSP73
ns
EV
RAB5A
35 kDa
SLUG
70 kDa
HSP73
***
25 kDa
RAB5A
15 kDa
Histone3
*
Protein expression relative to control
b
EV
RAB5A
Vimentin
Nuclei
