# Supplementary material for: RAB5A Promotes Active Fluid Wetting by Reprogramming Breast Cancer Spheroid Mechanics
Source: Adv Sci (Weinh). 2025 Jul 25;12(34):e03569. doi: 10.1002/advs.202503569 (PMC12442610; doi:10.1002/advs.202503569)
Supplement: Supplementary file 16 — Supporting Information [file ADVS-12-e03569-s005.pdf]

ADVANCED SCIENCE
Open Access

Supporting Information

for *Adv. Sci.*, DOI 10.1002/advs.202503569

RAB5A Promotes Active Fluid Wetting by Reprogramming Breast Cancer Spheroid Mechanics

*Grégoire Lemahieu, Paulina Moreno-Layseca, Tobias Hub, Carlo Bevilacqua, Manuel Gómez-González, Federica Pennarola, Federico Colombo, Andrew E. Massey, Leonardo Barzaghi, Andrea Palamidessi, Leon-Luca Homagk, Samuel F. H. Barnett, Alexander X. Cartagena-Rivera, Christine Selhuber-Unkel, Robert Prevedel, Xavier Trepat, Joachim P. Spatz, Johanna Ivaska, Giorgio Scita and Elisabetta Ada Cavalcanti-Adam**

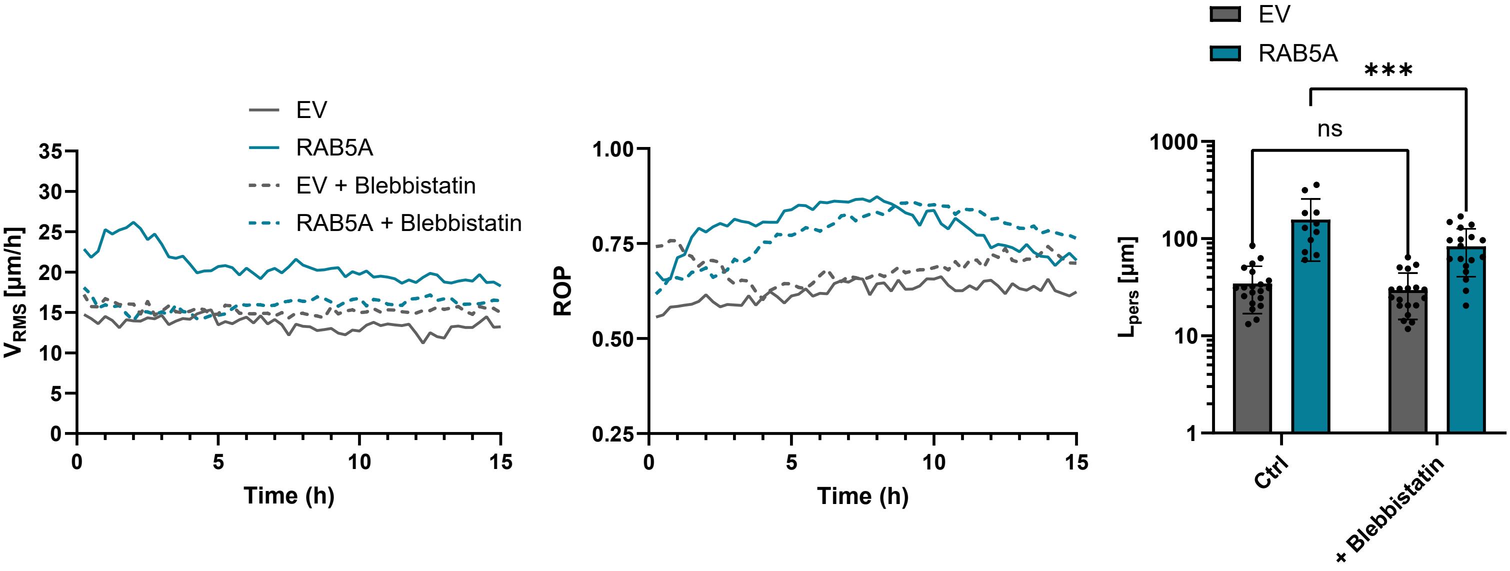

EV
RAB5A
EV + Blebbistatin
RAB5A + Blebbistatin
35
30
25
20
15
10
5
0
V_{RMS} [µm/h]
0
5
10
15
Time (h)
1.00
0.75
0.50
0.25
ROP
0
5
10
15
Time (h)
EV
RAB5A
***
ns
1000
100
10
1
L_{pers} [µm]
Ctrl
+ Blebbistatin
